# Inter- and intra-lineage genetic diversity of wild-type Zika viruses reveals both common and distinctive nucleotide variants and clusters of genomic diversity

**DOI:** 10.1080/22221751.2019.1645572

**Published:** 2019-07-29

**Authors:** Natalie D. Collins, Steven G. Widen, Li Li, Daniele M. Swetnam, Pei-Yong Shi, Robert B. Tesh, Vanessa V. Sarathy

**Affiliations:** a Department of Microbiology and Immunology, University of Texas Medical Branch, Galveston, USA; b Department of Biochemistry and Molecular Biology, University of Texas Medical Branch, Galveston, USA; c Department of Pathology, University of Texas Medical Branch, Galveston, USA; d Department of Pathology, Microbiology and Immunology, School of Veterinary Medicine at University of California, Davis, USA; e Sealy Institute for Vaccine Sciences, Institute for Human Infections and Immunity, University of Texas Medical Branch, Galveston, USA

**Keywords:** Next generation sequencing, Zika virus, genetic diversity, Zika lineage, Shannon entropy

## Abstract

Zika virus (ZIKV) strains belong to the East African, West African, and Asian/American phylogenetic lineages. RNA viruses, like ZIKV, exist as populations of genetically-related sequences whose heterogeneity may impact viral fitness, evolution, and virulence. Genetic diversity of representative ZIKVs from each lineage was examined using next generation sequencing (NGS) paired with downstream entropy and single nucleotide variant (SNV) analysis. Comparisons showed that inter-lineage diversity was statistically supported, while intra-lineage diversity varied. Intra-lineage diversity was significant for East but not West Africa strains. Furthermore, intra-lineage diversity for the Asian/American lineage was not supported for human serum isolates; however, a placenta isolate differed significantly. Relative entropy values were higher in the pre-membrane/membrane (prM/M) gene of several ZIKV strains. Additionally, the East African lineage contained a greater number of synonymous SNVs, while a greater number of non-synonymous SNVs were identified for American strains. Further, inter-lineage SNVs were dispersed throughout the genome, whereas intra-lineage non-synonymous SNVs for Asian/American strains clustered within prM/M and NS1 gene. This comprehensive analysis of ZIKV genetic diversity provides a repository of SNV positions across lineages. We posit that increased non-synonymous SNV populations and increased relative genetic diversity of the prM/M and NS1 proteins provides more evidence for their role in ZIKV virulence and fitness.

## GRAPHICAL ABSTRACT

**Figure:**
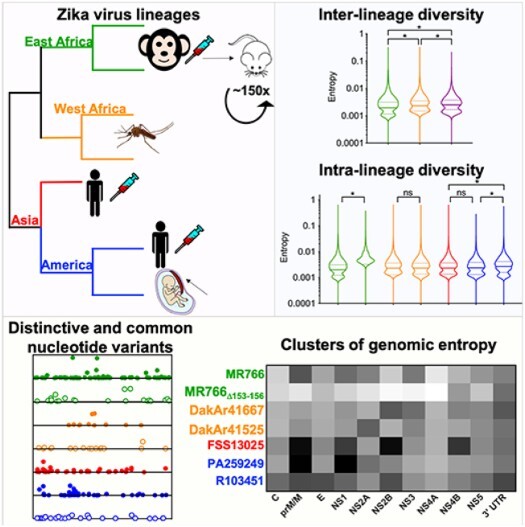

## Introduction

Zika virus (ZIKV) is a mosquito-borne flavivirus that was originally discovered in Uganda in 1947 after a sentinel Rhesus monkey developed fever and viremia [1]. Historically, human ZIKV infections occurred infrequently in Africa and Asia, and symptoms were limited to mild febrile disease [2]. In 2007, ZIKV infections on the island of Yap led to clinical signs including rash, fever, conjunctivitis, and arthralgia [3]. After initial spread out of Asia, ZIKV continued to cause clinical disease, including a 2013 outbreak in French Polynesia associated with Guillain-Barré syndrome [4,5]. Beginning in 2015, a ZIKV epidemic spread throughout the Americas, leading to foetal neurodevelopmental deficiencies, including microcephaly, termed Congenital Zika Syndrome [6].

ZIKV strains are grouped into three major phylogenetic lineages: East African, West African, and Asian/American [4,7–11]. Strains from the recent epidemic in the Americas form a clade within the Asian lineage.

ZIKV has a typical flavivirus genome organization of a positive-sense, single-stranded RNA of approximately 10,800 nucleotides in length. The genome encodes for a single polyprotein that is co- and post-translationally processed to generate ten viral proteins [12]. The three structural proteins: capsid (C), pre-membrane/membrane (prM/M), and envelope (E) have roles in viral maturation, entry and fusion with the host cell, and humoral immunity [13,14]. The seven nonstructural proteins: NS1, NS2A, NS2B, NS3, NS4A, NS4B, and NS5 encode for proteins involved in innate immunity, the replication complex, or viral enzymes, such as a helicase, methyl-transferase, or RNA-dependent RNA-polymerase (RdRp) [15]. Studies with flaviviruses of medical importance, such as dengue, West Nile, and Japanese encephalitis, have shown that molecular determinants of virulence are present throughout the genome [16–19].

RNA virus genomes exist as viral populations composed of genetically-related sequences. This is due, in part, to the error rate of the RdRp, which leads to nucleotide misincorporation during viral replication. As mutations accumulate, viral genomes can become more heterogeneous. Next generation sequencing (NGS) provides a platform to gain deeper understanding of viral diversity by increasing the depth of sequencing coverage, thereby allowing detection of single nucleotide variants (SNV) in the population. Evaluating genetic diversity has expanded the understanding of virulence, evolution, and host-specific adaptations. Flavivirus genetic diversity has been examined for the prototypical member, yellow fever virus (YFV). Comparisons between wild-type (WT) and live-attenuated YFV strains show that higher entropy is associated with a virulent, WT phenotype [20,21].

In the present study, NGS has been used to gain insight into the genetic diversity of representative ZIKV strains from different lineages. Intra-lineage diversity for Asian/American strains was not supported for human serum isolates; however, the diversity of a placenta isolate was significantly different. Furthermore, relative genomic diversity was highest in the prM/M and NS1 gene regions of Asian/American strains isolated from human serum. This study provides insight into the intra-host diversity of contemporary ZIKV that may contribute to mammalian infection or virulence.

## Materials and methods

### Viruses

ZIKV strains acquired from the World Reference Center for Emerging Viruses and Arboviruses (WRCEVA) (www.utmb.edu/wrceva) were chosen as representatives of the different genetic lineages from East Africa: MR766 and MR766_ΔE153-156_, from West Africa: DakAr41667 and DakAr41524, from Asia: FSS13025 and from the Americas PA259249 and R103451. Stocks were generated by passaging in monkey kidney Vero cells in minimum essential media (MEM) supplemented with L-glutamine, non-essential amino acids, penicillin–streptomycin, and 2% fetal bovine serum. Isolation and passage histories are described in Table 1 and selection details are provided in Supplementary Materials and Methods.

**Table 1.** Description of ZIKV strains used in study.

Lineage/clade	Strain	Country	Collection	Source	Passage history
**East Africa**	MR766	Uganda	Apr 1947	Sentinel Rhesus monkey (serum)	SM 151, Vero x2 / SM 151, Vero, C6/36, Vero
**East Africa**	(30306) MR766_ΔE153-156_	Uganda	Apr 1947	Sentinel Rhesus monkey (serum)	SM 149, Vero x2 / SM 150, Vero
**West Africa**	DakAr41667	Senegal	12 Jun 1984	*Aedes taylori* mosquito	AP61, C6/36 x3, Vero / AP61, C6/36 x3, Vero
**West Africa**	DakAr41524	Senegal	17 Nov 1984	*Aedes africanus* mosquito	AP61, C6/36 x2, Vero / AP61, C6/36 x2, Vero
**Asia**	FSS13025	Cambodia	10 Aug 2010	Human (serum)	Vero, C6/36 x2, Vero x2 / Vero, C6/36 x2, Vero x3
**America**	PA259249	Panama	11 Dec 2015	Human (serum)	Vero x3 / Vero x3
**America**	R103451	Honduras	6 Jan 2016	Human (placenta)	Vero x3 / Vero x3

SM: suckling mouse; C6/36: *Aedes albopictus* cells; AP61: *Aedes pseudoscutellaris* cells; Vero: African green monkey kidney cells. The passage histories of two replicate stocks are included and separated by (/).

### Phylogenetic analysis

Complete coding sequences of ZIKVs available from the WRCEVA were analysed using maximum-likelihood phylogeny. Nucleotide sequences were aligned with MUSCLE [22], and phylogeny was constructed using the RaXML-HPC Backbox tool [23] in the CIPRES Scientific Gateway using default parameters [24].

### Next generation sequence analysis

Viral RNA was sequenced at the UTMB Next Generation Sequencing (NGS) Core Facility using the Illumina platform. The analysis pipeline used in this study is described in Supplementary Materials and Methods and in Figure S1 according to the guidelines for computational reproducibility [25]. *De novo* consensus sequences were compared to reference sequences deposited in Genbank, with the exception of DakAr41667, for which there was no available sequence until this study (accession MF510857). Sequencing data are available in the ArrayExpress repository (accession E-MTAB-7771).

### Determination of diversity indices

Diversity indices were determined from sequence alignment data following removal of PCR duplicates. V-phaser2 v2.0, a software package for inferring low frequency variants, was used to determine variant population. All SNVs controlled for false discovery and strand-bias were assessed. The results in the form of a list of SNV positions, frequencies, and identities for each ZIKV strain were exported to Excel to facilitate data sorting and table organization. The amino acid identity encoded by each SNV was determined using the gene lengths in Table S3 and compared among the strains. Shannon entropy index was calculated to assess ZIKV genomic diversity. The variability (or uncertainty) at each nucleotide position from 107 to 10,700 was determined as previously described [26,27]. Briefly, deepSNV v1.16.0 bam2R with a quality score of 30 was used to determine nucleotide counts for genomic positions and then converted to relative nucleotide frequencies using RF. Shannon entropy was calculated utilizing local R script v3.2.4 (Figure S1) and relative nucleotide frequencies, according to previously reported analyses [28], with a maximum positional value of 1.61 indicating equal representation of all nucleotides (A, U, G, C, and gap). Data were evaluated for each strain (*n* = 2 each) or for each lineage (East Africa: *n* = 4, West Africa: *n* = 4, Asia/ America: *n* = 6). The inter- and intra-lineage entropy statistics were determined by combining the replicates for each strain within a lineage and non-parametric analysis.

### Structural renderings

Protein structures were rendered using Pymol v1.8.4.0: Capsid: PDB 5YGH, Membrane-Envelope: PDB 6CO8, NS1: PDB 5K6 K, NS3: PDB 5GJB, NS5: PDB 5UOB.

### Statistical analysis

Statistical tests (Mann–Whitney, Kruskal–Wallis and post-tests), correlations, (Spearman and Pearson) and calculations (mean, median, error) were determined using Prism v7.0 (GraphPad). Plots were generated using Prism v7.0 or 8.0.

## Results

### NGS generated a rich dataset to study genetic diversity

**Figure 1.**
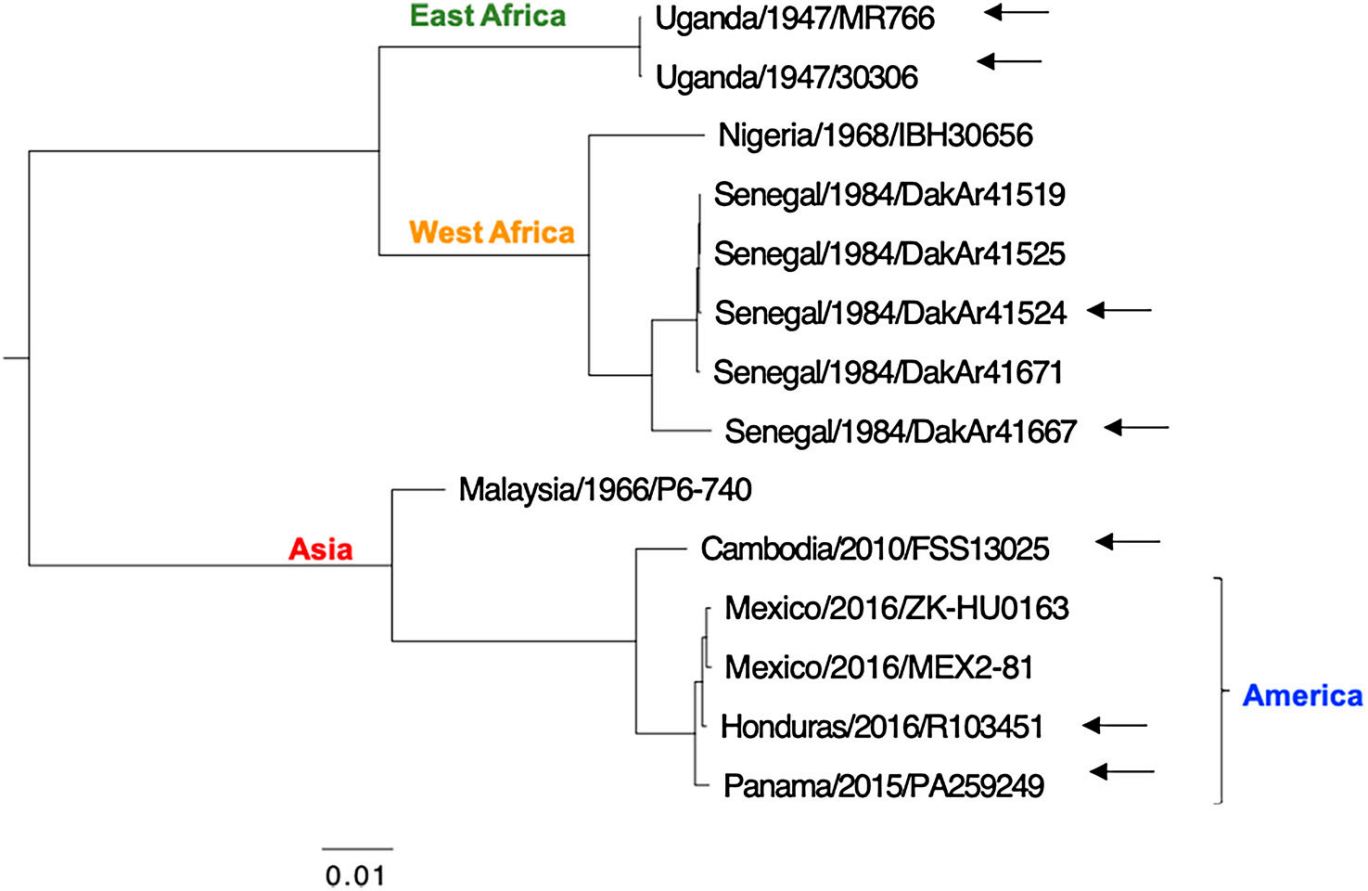
*Phylogenetic analysis.* Maximum-likelihood phylogeny of ZIKV strains from the WRCEVA supported the groupings into the East Africa, West Africa, and Asian lineages, the latter containing strains from the recent epidemic. Arrows point to the ZIKVs chosen for genetic diversity analysis.

Maximum likelihood phylogeny was constructed using ZIKV strains available from the WRCEVA (Figure 1). Tree topology was consistent with three lineages consisting of strains from East Africa, West Africa, and Asia and the Americas. Representative strains from each lineage were selected for genetic analysis (Table 1). The two East African strains originated from monkey serum in 1947 in Uganda [1]. Strain MR766 and strain MR766 stock 30306 (subsequently referred to as MR766_ΔE153-156_) differ in consensus sequences by four amino acids in the E protein (153-154 deletion), due to lengthy passage histories. Two West African strains, DakAr41524 and DakAr41667, were isolated in Senegal in 1984 from *Aedes africanus* and *Ae. taylori* mosquitoes, respectively. Next, three Asian/American lineage strains were selected for the study. Strain FSS13025 was isolated in Cambodia in 2010 from the acute phase serum of a child [29]. Strain PA259249 was isolated from human serum in Panama in December 2015. Lastly, a human placenta isolate, strain R103451, was isolated in Honduras in January 2016. Strains PA259249 and R103451, have an identical low number of passages in Vero cells.

**Figure 2.**
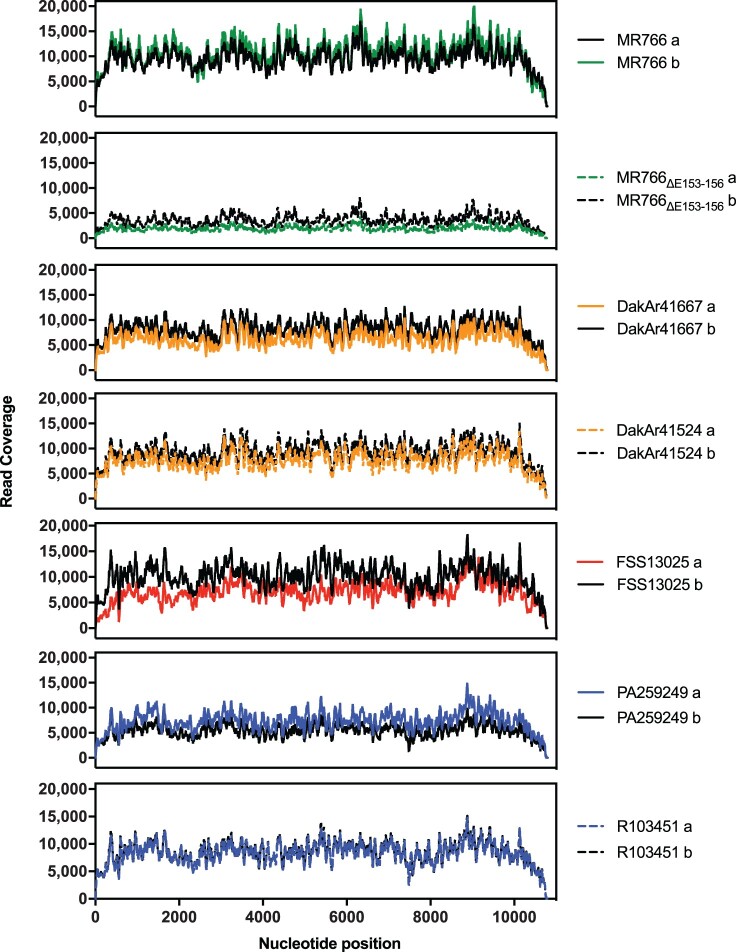
*ZIKV NGS sequencing dataset description.* Duplicate samples (*N *= 14) of each strain were analysed. The positional read coverage depth of each replicate (after filtering out low-quality reads and PCR replicates) was plotted along the genome and was similar for both replicates of each strain. Coverage range: 1,906-10,762.

Two biological replicates of the seven ZIKVs were subjected to NGS and downstream analysis. Mapped reads ranged from 415,219–2,526,545. After processing the mean map quality of the strains was approximately 44 (Table S1), and the mean depth of coverage across the genome ranged from 1,906–10,762, with consistent positional coverage for both replicates of each strain (Figure 2). Mean genome coverage and mapped reads were highly correlated (*r *= 0.9961) (Figure S2). *De novo* consensus sequences for strains MR766, MR766_ΔE153-156_, DakAr41524, FSS13025, PA259249, and R103451 were aligned to the corresponding publicly available sequences using MUSCLE, which showed that some *de novo* sequences were missing up to 10 nucleotides from the 5’ and/or 3’ ends, so the sequences were adjusted with N placeholders accordingly. Additionally, FSS13025 and R103451 each contained the amino acid substitutions K1059E and K733R, respectively, compared to published sequences (Table S2). Overall, the NGS results provided a rich dataset to perform downstream genetic analysis.

### Inter- and intra-lineage comparisons reveal differences among Asian/American lineage ZIKVs

To determine the nucleotide diversity of ZIKVs, Shannon entropy was computed. This calculates the nucleotides at a given position, and higher values indicate that all nucleotides are equally represented at that position. To account for bias introduced from lower depths of coverage at the 5’ and 3’ ends of the genome, Shannon entropy was calculated for every position starting at nucleotide 107 (the A of the first codon in African lineage strains) through nucleotide 10,700, approximately 100 nucleotides from the 3’ terminus of the ZIKV genome and the mean of the two replicates was determined (Figure S3).

**Figure 3.**
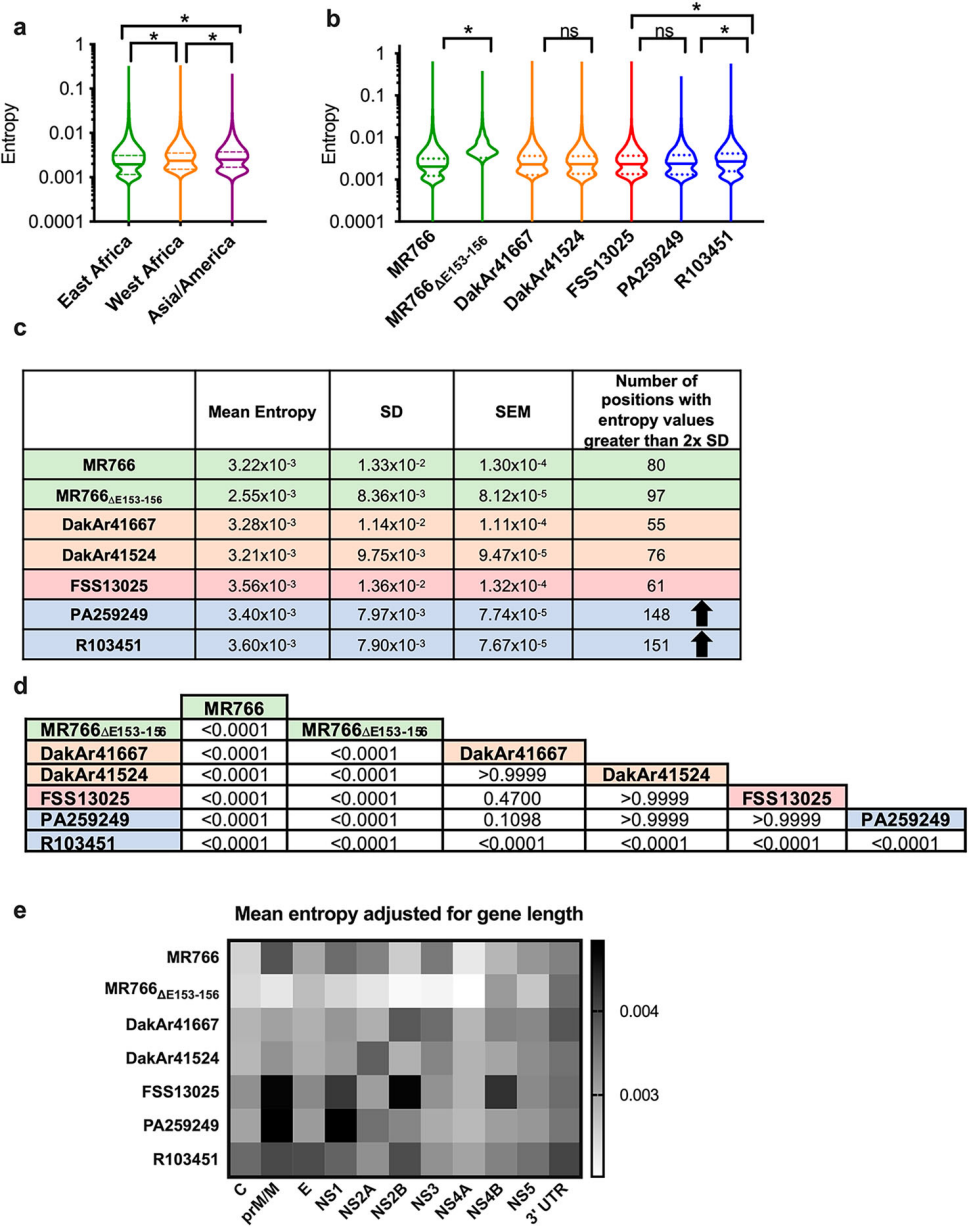
*Shannon entropy analysis shows inter- and intra-lineage genetic diversity relationships.* The mean Shannon entropy calculated from positions 107-10,700 from two biological replicates of each strain for the East Africa (*n* = 4), West Africa (*n* = 4), and Asia/America (*n* = 6) lineages (A) or by strain (each, *n* = 2) (B) is shown as violin plots; solid lines: median, dashed lines: interquartile range, statistics determined using Kruskal-Wallis with Dunn’s post-test. C) Mean entropy values for each strain (*n* = 2) with standard error of the mean (SEM) and standard deviation (SD). The number of positions with entropy values greater than two times the SD are shown; arrows denote that strains PA259249 and R103451 contain more high entropy positions. D) Statistical analysis (Kruskal-Wallis with Dunn’s post-test) of mean genomic Shannon entropy comparing (*n* = 2) of each strain. E). Relative diversity heat map shows the mean Shannon entropy of each gene region relative to each gene length.

Inter-lineage nucleotide diversity was assessed by grouping the strains phylogenetically prior to performing statistical analysis (Figure 3a). Results show that East African, West African, and Asian/American lineages differ in genomic nucleotide diversity (Kruskal–Wallis, Dunn’s post-test, *p *< 0.0001), with mean entropy values of 2.88 × 10^−3^, 3.24 × 10^−3^, and 3.52 × 10^−3^, respectively, indicating increased nucleotide diversity for the Asian/American lineage.

To assess within-lineage diversity, entropy was calculated for each strain. The intra-lineage diversity is statistically significant (Kruskal–Wallis, Dunn’s post-test) for strains of the East African lineage (*p *< 0.0001) and for strains of the Asian/American lineage (*p *< 0.0001) but not for strains of the West African lineage (Figure 3b-d). Interestingly the diversity of human placenta strain R103451 was significantly different (*p *< 0.0001) to that of both strains FSS13025 and PA259249, but diversity of the latter two was not significantly different to each other.

The mean nucleotide diversity for the ZIKVs ranged from 2.56 × 10^−3^–3.60 × 10^−3^ (Figure 3c). MR766 has higher mean genomic entropy than MR766_ΔE153-156_, indicating that passaging of the latter led to both a sequence deletion and lower genomic diversity. The contemporary strains (Asian/American) had increased genomic diversity compared to strains from Africa. Specifically, strain R103451 had the highest mean entropy in the dataset. Next, high diversity values for nucleotide positions in a given strain were defined as those with entropy values greater than 2σ (two times the standard deviation) of the Shannon entropy of the population for each strain. The number of nucleotide positions containing high nucleotide diversity was greater for strains PA259249 and R103451 (148 and 151, respectively), accounting for 1.4% of the positions examined, but for other strains, high entropy positions (range of 55-97) comprised less than 1% of the positions (Figure 3c and S3). Overall, strains PA259249 and R103451 contained 1.7-, 2.3-, and 2.5-fold greater numbers of high diversity positions than East African, West African, or Asian (FSS13025) (*p *= 0.066), respectively.

Shannon entropy of each gene region (Table S3) was analysed to determine the distribution of genetic diversity. Cumulatively, genetic diversity corresponded to the gene length (Figure S4), with the NS5 gene containing highest entropy values, followed by NS3, E, and NS1. However, relative diversity, expressed relative to gene length, had a different distribution (Figure 3e). The two human serum strains exhibited highest relative diversity in many gene regions compared to other strains. Specifically, the prM/M and NS2B gene region were highest for strain FSS13025, and prM/M and NS1 gene regions were highest for strain PA259249 (Figure 3e). These results indicate that high relative genetic diversity may be important for the role of the prM/M during infection.

### Single nucleotide variant sites are distributed throughout the ZIKV genome

**Figure 4.**
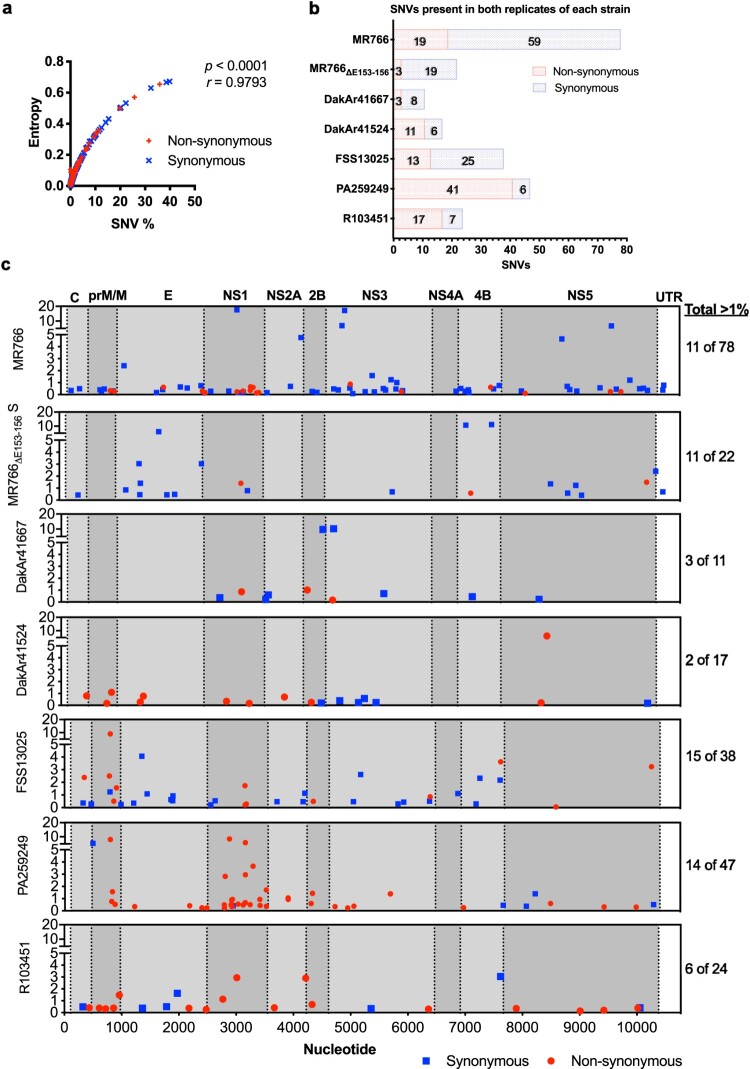
*SNVs detected in all ZIKV strains.* A) For all positions with SNVs, the frequency percentage and entropy values are correlated (Spearman correlation, *r *= 0.9793). B) The total number of SNVs identified and common to both replicates of each ZIKV are shown: synonymous change (blue bar), non-synonymous change (red bar). C) Mean frequency values of all SNVs common to both replicates were plotted by nucleotide position, synonymous (blue), non-synonymous (red). Genomic regions (UTRs and proteins) are denoted by dotted lines and alternating background shading under a genome map. The number of SNVs present at greater than 1% frequency out of the total number of SNVs is provided on the right.

The contribution of SNVs to the genetic population of ZIKVs was determined using Vphaser2 v2.0. Results from duplicate datasets were combined to yield a total number of SNV positions ranging from 44 to 170 depending on the strain. SNVs were categorized as encoding synonymous or non-synonymous substitutions (Tables S4 and S5, respectively), and comprehensive lists are provided in the supplement. The frequency of each SNV was plotted against the corresponding positional entropy value to reveal a strong correlation between entropy value and SNV frequency (Figure 4a). Subsequent analysis was performed only with SNVs that were present in both biological replicates of each ZIKV. Strains MR766, MR766_ΔE153-156_, and DakAr41667, and FSS13025 have greater number of synonymous SNVs, whereas strains DakAr41524, PA259249, and R103451 have greater numbers of non-synonymous SNVs (Figure 4b).

SNV frequency was plotted along the genome, and all strains had SNVs exceeding 1% frequency of the population (Figure 4c). Of these, a majority were non-synonymous for American strains: PA259249 (12 of 14) and R103451 (4 of 6), but not for the others: MR766 (0 of 11), MR766_ΔE153-156_ (2 of 11), DakAr41667 (1of 3), and DakAr41524 (1of 2), and FSS13025 (7 of 15).

Several non-synonymous SNVs with greater than 1% frequency were located in the prM/M gene encoding for: DakAr41524 A136; FSS13025 K104, S109, L145; PA259249 T107, T119; and R103451 I163. Furthermore, Asian/American strains had several non-synonymous SNVs with greater than 1% frequency in the NS1 protein: FSS13025 M220; PA259249 R103, K128, M220 (2 nucleotides), K265, and E342; and R103451 K94 and Y175. Surprisingly, strain PA259249 contained 21 SNVs in the NS1 gene; 20 were non-synonymous, and 6 were present at a frequency of 1% or greater. Furthermore, strains MR766, FSS13025, and PA25924 contained multiple SNVs at a given amino acid position (Table 2). Most were located in the NS genes, specifically in NS1. These data showed a trend for flexible codon usage in the NS1 gene, particularly for strain PA259249, suggesting it possesses a high degree of variant diversity.

**Table 2.** ZIKV amino acid positions containing multiple variants in the same codon.

ZIKV	Position	%	Synonymous / non-synonymous^1^	Codon position	Consensus amino acid	Variant amino acid	Polyprotein
MR766 NS1 271	G3299A	**0.361**	N	1	E	K	1065
MR766 NS1 271	A3300G	**0.769**	N	2	E	G	1065
MR766 NS2A 15	G3589T	**0.168**	S	3	V	V	1161
MR766 NS2A 15	G3589A	0.239	S	3	V	V	1161
MR766 NS3 156	T5079A	0.035	N	2	I	N	1658
MR766 NS3 156	C5080A	0.043	S	3	I	I	1658
MR766 NS3 156	C5080T	0.111	S	3	I	I	1658
FSS13025 NS1 220	A3147G	**1.877**	N	1	M	V	1014
FSS13025 NS1 220	G3149T	**0.247**	N	3	M	I	1014
FSS13025 NS1 220	G3149A	1.396	N	3	M	I	1014
FSS13025 NS5 855	C10230T	**3.301**	N	1	H	Y	3375
FSS13025 NS5 855	C10232T	0.251	S	3	H	H	3375
PA259249 prM/M 135	T876C	**0.797**	N	1	F	L	257
PA259249 prM/M 135	C878A	0.344	N	3	F	L	257
PA259249 NS1 98	T2781C	**0.611**	N	1	W	R	892
PA259249 NS1 98	G2782C	0.153	N	2	W	S	892
PA259249 NS1 98	G2783T	**0.243**	N	3	W	C	892
PA259249 NS1 220	A3147G	**0.903**	N	1	M	V	1014
PA259249 NS1 220	T3148C	**3.662**	N	2	M	T	1014
PA259249 NS1 220	G3149A	**10.23**	N	3	M	I	1014
PA259249 NS1 304	A3399G	0.293	N	1	S	G	1098
PA259249 NS1 304	G3400A	**1.302**	N	2	S	N	1098
PA259249 NS1 342	G3513A	**0.426**	N	1	E	K	1136
PA259249 NS1 342	A3514G	**1.880**	N	2	E	G	1136
PA259249 NS2A 117	G3894A	**1.101**	N	1	A	T	1263
PA259249 NS2A 117	C3895T	**1.193**	N	2	A	V	1263

Synonymous (S), non-synonymous (N) variants within amino acid.

Mean variant frequency % of two duplicates is shown in bold type.

### Common inter- and intra-lineage SNVs

**Figure 5.**
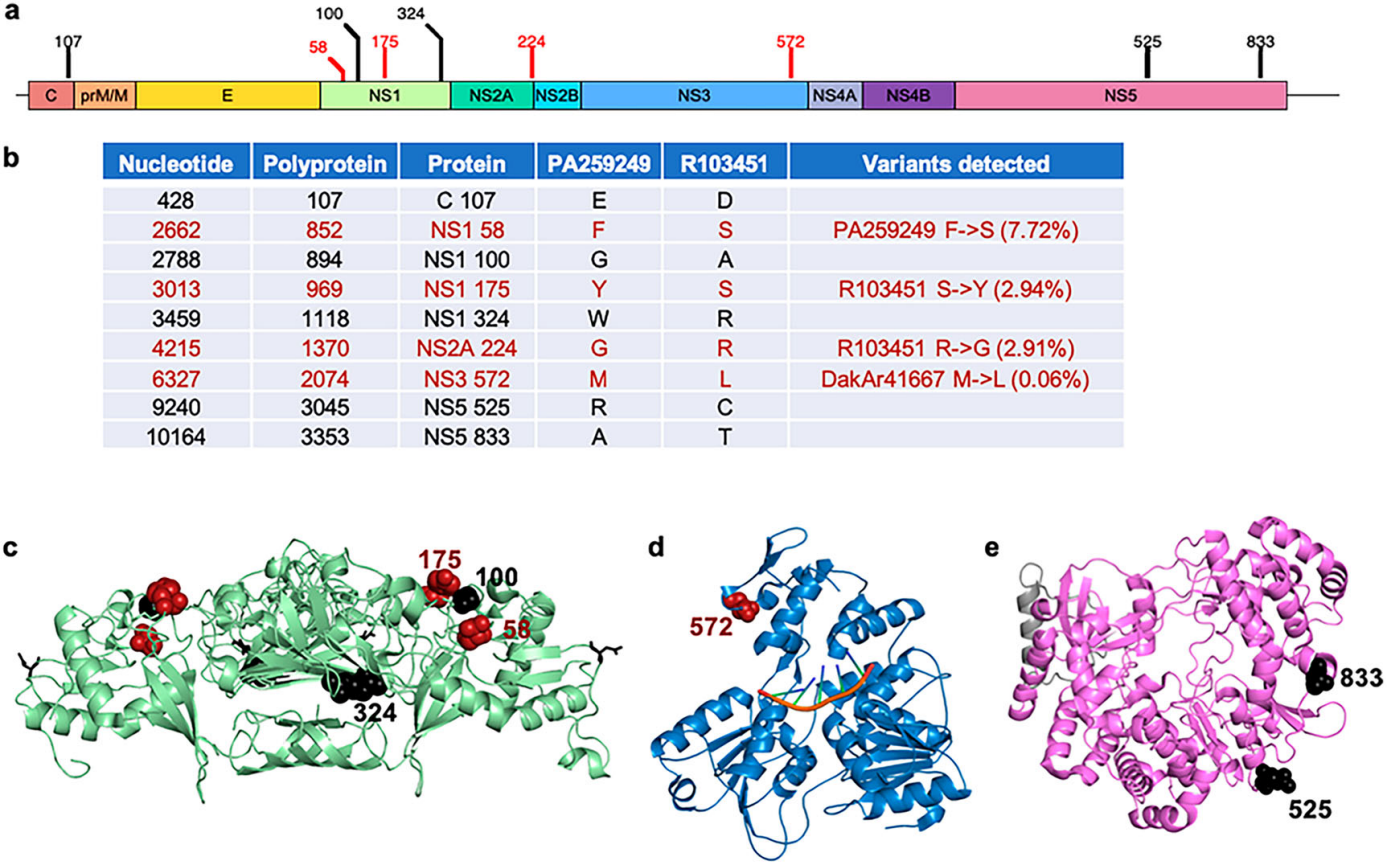
*SNVs were detected in four of the nine consensus sequence changes between strains PA259249 and R103451.* A) Genome schematic depicting the amino acid differences between strains PA259249 and R103451, red: denotes positions of SNVs in the dataset across lineages. B) Nucleotide, polyprotein, and protein number of coding differences between the two strains, as well as the variants detected (% denotes frequency of SNV) are listed. Structural depiction of each consensus coding change, in black, or consensus coding change with SNV detected, in red, for the NS1 dimer (C), NS3 helicase (D), and NS5 polymerase (E). No structure is available for amino acids C 107 and NS2A 224.

In order to discover advantageous SNVs that were maintained in the population, variants shared by one or more ZIKV strains were identified (Table 3 and Figure 5). Fourteen of the seventeen SNVs shared by at least two ZIKVs are located in ZIKV proteins with solved structures: C, (prM/)M, E, NS1, NS3, and NS5 proteins (Figure S5). No SNVs were shared between East and West African lineages. However, the Asian/American lineage shared 11 SNV amino acid positions with either the East or West African lineages (Table 3). Seven SNV positions were shared between the East African and Asian/American lineages: C G73, prM/M F130, E A270, NS1 L145, NS1 K265, NS4B F86, and NS5 H855. Two SNVs, NS1 L145 and NS1 K265, were less than 1% and synonymous in the East African lineage but greater than 1% and non-synonymous in Asian/American lineage. Four SNV positions were shared between the West African and Asian/American lineages: prM/M S109, NS1 M220, NS2A A117 and NS5 C269. Three non-synonymous SNVs (prM/M S109, NS1 M220 and NS2A A117). were present at greater than 1% frequency in Asian/American strains yet were less than 1% frequency in the West African Strains.

**Table 3.** Common inter- and intra-lineage SNVs per protein amino acid position.

Protein	Polyprotein #	MR766	MR766_ΔE153-156_	DakAr41667	DakAr41524	FSS13025	PA259249	R103451	#lineages
**C 73**	73	G->G (0.53)	G->G (0.61)			G->G (0.47)		G->G (0.53)	2
**prM/M 107**	229*					T->T (1.37)	T->M (9.42)		1
**prM/M 109**	231				S->P (0.18)	S->P (11.55)			2
**prM/M 130**	252	F->L (0.39)				F->L (0.65)		F->L (0.42)	2
**E 270**	560	A->V (0.79)						A->A (0.54)	2
**E 401**	691*						H->Y (0.45)	H->Y (0.41)	1
**E 501**	791*						A->T (0.25)	A->T (0.30)	1
**NS1 145**	939	L->L (0.30)					L->P (1.07)		2
**NS1 220**	1014			M->T (0.87)		M->V (1.88) M->I (1.40)	M->V (0.90) M->T (3.66) M->I (10.23)		2
**NS1 228**	1018/1022	S->P (0.40)	S->P (2.08)						1
**NS1 265**	1055/1059		K->K (0.93)				K->E (4.13)		2
**NS2A 117**	1263				A->V (0.89)		A->V (1.19) A->T (1.10)		2
**NS3 583**	2085*					R->R (0.75)		R->K (0.36)	1
**NS4B 86**	2351/2355		F->L (0.61)			F->F (0.36)			2
**NS4B 206**	2471/2475	L->L (0.54)	L->L (12.47)						1
**NS5 269**	2789				C->R (6.33)		C->R (0.61)		2
**NS5 855**	3371/3375		H->Y (1.72)			H->Y (3.30) H->H (0.25)			2

*Shared by Asian/American strains only.

( ) Mean variant frequency % of two duplicates.

Intra-lineage comparisons revealed two SNV positions common to only East African strains (NS1 S228 and NS4B L206) and four SNV positions common to only Asian/American strains (prM/M T107, E H401, E A501, and NS3 R583) (Table 3). The high frequency SNV prM/M T107 was only shared between strains FSS13025 and PA2592495. It was a synonymous, codon position 3, for strain FSS13025 but non-synonymous, codon position 2, for strain PA259249 (Tables S4 and S5). Likewise, strains R103451 and FSS13025 both had a SNV in NS3 583: synonymous, codon position 3, in strain FSS13025 but non-synonymous, codon position 2, in strain R103451. All of the SNVs shared between PA259249 and R103451 were low frequency SNVs. Several shared SNVs were located in the carboxy-terminus of the E protein and the transmembrane portion of the cleaved prM/M protein located in the mature virion (Figure S5b). SNVs shared in the NS1 protein localized to the beta-ladder (220, 228, 265) and to the wing domain (145). Together, these data demonstrate that specific SNVs are maintained in the genomic populations of ZIKVs among lineages.

### Comparison of strains from the Americas

The genomes of the two American strains were examined in detail in order to compare the diversity of two strains with identical passage history. The mean genomic entropy was higher for strain R103451 (Figure 3c). However, strain PA259249 had approximately twice the number of SNVs. Approximately 25% of SNVs from both strains exceeded 1% of the population (Figure 4c). Strains PA259249 and R103451 differ in consensus genomic sequence by 38 nucleotides and 9 amino acid substitutions throughout the genome (Figure 5), 4 of which were located in the NS1 protein (F58S, G100A, Y175S, and W324R). SNVs were detected for two of these changes in the dataset, F58S (7.72%) in strain PA259249 and S175Y (2.94%) in strain R103451. Additionally, the consensus change NS2A G224R was also a SNV in R103451 (2.91%). Lastly, the consensus change NS3 M572L was a SNV detected in DakAr41667, albeit at a low frequency (0.06%). These data take into account the similarity in cell culture passaging of the two strains from the Americas to reveal differences in genomic diversity.

### Analysis of NGS data from the public repository

A limited set of NGS data deposited in Sequence Read Archive (SRA) was analysed in order to corroborate the genetic diversity identified in this study with existing sequence data. Public data for strains MR766_ΔE153-156_, DakAr41524, PA259249, and R103451 were analysed using the pipeline described in this study to determine the SNVs in each sample (Figure S6). Because the deposited sample data contained 4–10 runs for each sample entry, the data were pooled during analysis. However, the mean coverage across the genome was still lower in the downloaded dataset, with a range of 332-1,611 after processing. Strain PA259249 (sample #1) was the only sample with coverage >1500, which is the cut-off typically used for downstream entropy and SNV analysis. Nevertheless, a SNV analysis was performed. For MR766_ΔE153-156_ (#4) the overwhelming majority of SNVs were synonymous (Figure S6), as previously characterized (Figure 4), whereas the majority of SNVs for strains PA259249 (#1) and R103451 (#5) were non-synonymous (Figure S6), as previously characterized (Figure 4). However, analysis of two different entries of strain DakAr41524 (#2 and #6) resulted in different numbers of SNVs (64 and 3, respectively). The number of SNVs that were shared among the stocks sequenced in this study and the downloaded data are listed in Table S4 as follows: MR766_ΔE153-156_ (#4)−4; DakAr41524 (#2)−3; DakAr41524 (#6)−1; PA259249 (#1)−18; and R103451 (#5)−5. Notably NS1 145, NS1 220, and NS2A 117 were shared among several strains, as previously characterized (Table 3 and S6). Overall, analyses of the sequence data downloaded from the SRA and the data generated in this study had similaries. However, some strains differed in the SNVs, possibly due to differences in sample passages, sequencing platforms, genome coverage depth, and number of runs. These factors can influence genetic diversity studies.

## Discussion

The present study evaluated genetic diversity across lineages of ZIKV analysing NGS of representative strains from various sources (mosquito and mammalian), including a human placenta isolate. The dataset was robust with sufficient coverage and map quality scores (Figure 2, Table 2) to analyse intra-host diversity of ZIKV. The mean genomic diversity of the Asian/American lineage was higher than those of the African lineages, and American isolates had a greater number of high entropy positions (Figure 3a-c and S3). Intra-lineage diversity differed between the East African strains MR766 and MR766_ΔE153-156_, likely due to the number of passages since isolation in 1947. However, there was no statistical support for intra-lineage diversity difference between the two West African strains, whose passage histories differed by one passage (Table 1). The Asian/American lineage strains FSS13025 and PA259249 were not statistically different in diversity, yet the diversity of strain R103451 differed significantly from both. Because both strains PA259249 and R103451 were passaged similarly, the intra-host diversity differences may be due to other characteristics, such as the geographic location of isolation (Panama versus Honduras) or to tissue-specific (serum versus placenta) effects on the genomic diversity of ZIKV. Other studies have shown that the genetic diversity of serum samples collected during the outbreak in the Americas does not differ from that of Asian strains [30,31], which supports the data in this study. One limitation of this study is that, due to widespread isolation times and locations, the ZIKV strains selected were passaged a number of times in different cells or tissues which may not reflect the natural diversity of these ZIKV strains. For each ZIKV, the lowest passage possible was used. It is worth noting that strains FSS13025 and PA259249 did not differ in diversity even though the former was passaged twice in C6/36 mosquito cells, while the latter was not passaged in C6/36 cells at all.

Amino acids in prM/M and NS1 proteins have been associated with virulence of ZIKV strains [32–34]. The present analysis showed that Asian/American strains (and strain MR766) had higher relative entropy values in the prM/M gene region, and additional regions of high relative diversity for the Asian/American lineage include NS1 (FSS13025, PA259249, and R103451), E (R103451), NS2B (FSS13025 and R103451), NS4B (FSS13025), and 3’UTR (R103451). Therefore, the clusters of highest genomic diversity for the Asian/American lineage are located in the prM/M and NS1 gene regions.

Sub-consensus-level diversity was also measured by SNV analysis. Strain MR766 contained a greater number of SNVs than all other strains, especially compared to MR766_ΔE153-156_, which suggests that passaging of the latter led to not only a deletion in the E protein, but also decreased the SNV population. Interestingly, the mosquito isolates from West Africa had the fewest number of high frequency SNVs. The possibility that this is due to a bottleneck caused by virus propagation in arthropod cells followed by mammalian cells cannot be excluded; however, molecular epidemiology showed that contemporary (American) isolates of human serum, urine, or plasma clustered together with those from mosquito pools from the Americas [30], which may or may not be extrapolated to other ZIKV lineages. The Asian/American lineage strains had four non-synonymous SNVs in the prM/M gene region and at least two in the NS1 gene region. Most notably, strain PA259249 had 20 non-synonymous SNVs in NS1, which was the greatest number of non-synonymous SNVs per gene region of the dataset (Figure 4c). Lastly, inter-lineage SNVs were dispersed throughout the genome.

Several SNVs detected in this study correspond to positions of evolutionary importance identified in phylogenetic studies. Specifically, 17 of the amino acid changes reported by Pettersson and colleagues [11] contained SNVs in at least one strain replicate in the present study (Tables S4 and S5), and several were located in both replicates of at least one strain: C 110 (R103451), NS3 583 (FSS13025 and R103451) and NS5 784 (R103451). These three amino acids and nine more were also reported by Wang and colleagues [35]. Ultimately, sequence analyses can work in concert to provide insight into the composition and genetic diversity of ZIKVs.

Previous variant analyses of ZIKVs from different sources are in accordance with the present study. NGS analysis of ZIKV isolated from macaques experimentally infected with the French Polynesia strain led to identification of eight variants with frequencies greater than 5%, most of which were located between the carboxy-terminus of the E protein through NS2B [36]. Likewise, NGS analysis of WT strain PRVABC59 amplicons showed that the majority of variants are located in the E and NS1 protein regions [37]. Several NGS analyses of American ZIKV strains have examined the molecular epidemiology of ZIKV during the recent epidemic [30,31,38]. The sequences of 110 patient and mosquito samples resulted in identification of 21 SNVs, the majority of which (18) occurred in nucleotides 118-3,987 (spanning from C to NS2A protein) [30]. Five of those 21 SNVs were detected in the present study, and three additional SNVs were detected in the same codon (but not nucleotide position) [30], which provides additional evidence for clusters of diversity.

A previous study evaluating similar ZIKV strains identified consensus differences within and between lineages and conducted an extensive phylogenetic analysis, while the variant analysis was restricted to high frequency SNVs. Interestingly, five SNVs identified by Shrivastava and colleagues were also identified in this study (prM/M 46, E 161, E 401, NS2A 117, and NS2A 157), albeit in lower frequencies and not necessarily in the same strain. The differences in number, strain, and frequencies are likely due to the combination of different variant analysis software used and the restriction to high frequency SNVs. Vphaser2, used in the present study, was validated with a Flavivirus dataset and designed to identify low frequency SNVs, thus allowing analysis of all identified SNVs [39].

Although, the present study focused on genetic analysis, examination of these isolates *in vitro* and *in vivo* in the literature has shown that ZIKVs belong to a single serotype and share a highly virulent phenotype in immunocompromised mice, while NHPs recover from infection. Strains MR766 and FSS13025 have been characterized in immunocompromised mouse [40–42] and non-human primate (NHP) models [43–46]. An early study in NHPs found that the E protein non-glycosylated mutant (equivalent to MR766_ΔE153-156_) regained the glycosylation site following *in vivo* infection [43]. Strain DakAr41667 is also virulent in immunocompromised mice [40]. Strains PA259249 and R103451 have been used for baby mouse challenge [47] and *in vitro* analyses [48,49]. Lastly, the virulence of strain DakAr41524 has been shown to be comparable to that of MR766 in dendritic cells [50].

This study provides a broad view of ZIKV genetic diversity and posits that recent American isolates harbors increased non-synonymous SNV populations that may contribute to the virulence of contemporary ZIKVs in humans. The present analyses did not include phenotypic studies; however, studies have shown that both prM/M and NS1 may contribute to fitness or virulence. The prM/M S17N substitution increased infection of neural cells and neonatal mice, with the latter exhibiting increased mortality. Also, embryonic infection of mouse brains with virus encoding prM/M S17N led to microcephaly, including smaller brain size and increased apoptosis in brain cortices [33]. Also, a recent study found that the prM/M protein has a role in neural cell cytotoxicity and apoptosis *in vitro* [51]. Vaccine studies showed that inclusion of the prM/M sequence in an adenovirus-vectored vaccine containing the E protein increased the ZIKV-specific neutralizing antibody response and provided sterilizing immunity in a mouse challenge model [52]. These results suggested that interactions between prM/M and E are important for epitope display in ZIKV. Furthermore, subsequent studies found that presence of both prM/M and NS1 in the adenovirus-vectored vaccine was important for effective protection in mouse models because an Ad2-prM-E-NS1 vaccine provided better protection to neonatal mice than Ad2-prM-E [53]. Moreover, an evaluation of a prM-E-NS1 recombinant VSV-vectored vaccine showed that NS1 likely plays a role in modulating the cellular immune response during vaccination [54]. Furthermore, NS1 V188A was implicated in antagonism of IFN-beta induction by decreasing the phosphorylation of the TBK1 intermediate [32]. The NS1 V188A mutation also increased infectivity in *Aedes aegypti* mosquitoes [34].

The American ZIKV strain PRVABC59 was originally isolated from human serum. A recent study showed that Vero cell passaging led to increased emergence of the variants NS1 W98G and E V330L, presumably to increase fitness in Vero cell culture. The presence of both SNVs attenuated strain PRVABC59 in mice [55]. In the present study, SNVs were also detected at NS1 W98 in all three codon positions of strain PA259249; however, the resulting substitutions differed W98R/S/C and were low frequency (0.2-0.5%). Another NS1 mutation, K265E, increased ZIKV replication in Vero cells by increasing virion assembly; addition of a second mutation at prM/M H83R further increased viral titers in Vero cells [56]. Both of these amino acids were detected as SNVs in the present analysis. The SNV at NS1 K265E was detected in both DakAr41524 (0.2%) and in PA259249 (3.7%), while a synonymous SNV (0.8%) at this position was detected in MR766_ΔE153-156_. Lastly, prM/M H83R was identified as a SNV (0.3%) in R103451.

The present study shows a clustering of non-synonymous SNVs in the prM/M and NS1 proteins, especially in the contemporary ZIKVs. These results are in accordance with previous ZIKV characterizations in the literature. Subsequent analyses will be required in order to determine the contribution of SNVs to virulence and fitness.

## Supplementary Material

Supplemental Material
