# Supplementary material for: Inter- and intra-lineage genetic diversity of wild-type Zika viruses reveals both common and distinctive nucleotide variants and clusters of genomic diversity
Source: Emerg Microbes Infect. 2019 Jul 29;8(1):1126–38. doi: 10.1080/22221751.2019.1645572 (PMC6711133; doi:10.1080/22221751.2019.1645572)
Supplement: Supplemental Material [file TEMI_A_1645572_SM6046.docx]

**SUPPLEMENTARY INFORMATION**

Inter- and intra-lineage genetic diversity of wild-type Zika viruses reveals both common and distinctive nucleotide variants and clusters of genomic diversity

Natalie D. Collins^1^ [natalie.d.collins.mil@mail.mil](mailto:natalie.d.collins.mil@mail.mil), Steven G. Widen^2^ [sgwiden@utmb.edu](mailto:sgwiden@utmb.edu), Li Li^3^ [lili@utmb.edu](mailto:lili@utmb.edu), Daniele M. Swetnam^4^ [Dmswetnam@ucdavis.edu](mailto:Dmswetnam@ucdavis.edu), Pei-Yong Shi^2^ [peshi@utmb.edu](mailto:peshi@utmb.edu), Robert B. Tesh^3^ [rtesh@utmb.edu](mailto:rtesh@utmb.edu), Vanessa V. Sarathy^3,5^* [vvsarath@utmb.edu](mailto:vvsarath@utmb.edu).

^1^Department of Microbiology and Immunology, University of Texas Medical Branch, Galveston, TX, USA, ^2^Department of Biochemistry and Molecular Biology, University of Texas Medical Branch, Galveston, TX, USA, ^3^Department of Pathology, University of Texas Medical Branch, Galveston, TX, USA, ^4^Department of Pathology, Microbiology and Immunology, School of Veterinary Medicine at University of California, Davis, CA, TX, USA, ^5^Sealy Institute for Vaccine Sciences, Institute for Human Infections and Immunity, University of Texas Medical Branch, Galveston, TX, USA.

**Supplementary Materials and Methods**

***ZIKVs analysed:*** Strains from each lineage/clade were chosen to represent East Africa, West Africa, and Asia/Americas. The East African strains have been subjected to approximately 150 passages in suckling mice prior to conventional cell culture passages and are considered to be mouse-adapted. It was observed that both of the Uganda strains had identical sequences with the exception of only four amino acids in the E glycosylation site. The two strains are routinely used in ZIKV research and Aliota and colleagues [22] showed that the glycosylated form of MR766 could be recovered from NHP tissues following infection with the non-glycosylated MR766 (MR766_∆E153-156_); both were selected for downstream analysis. Next, because the Dakar strains were all isolated from *Aedes* mosquitoes in 1984, only two were chosen for analysis. Lastly, strains FSS13025, PA259249, and R103451 were chosen to represent the contemporary isolates due to the content of ZIKV sequences in the samples and their short passage history. Specifically, the only two strains with the exact same passage history were PA259249 and R103451, and an extended comparison of those two strains is included in this study. Following sequencing, some strains had poor sequencing quality results. Therefore, only replicates with sequencing coverage greater than 1,500 were analyzed.

***Next generation sequence analysis*:** Viral RNA from two separate virus stocks per strain was harvested using the Viral RNA isolation kit (Qiagen) and sequenced at the UTMB Next Generation Sequencing (NGS) Core Facility. cDNA libraries were constructed using random hexamers with the TruSeq RNA v2 kit (Illumina) and sequenced on an Illumina HiSeq1500 instrument. The next generation sequencing pipeline (Figure S1) used to process and analyze the dataset was composed of open source interfaces and packages, unless otherwise stated, and were installed directly or with HomeBrew, a package manager (https://brew.sh). Raw files containing pair-end reads were trimmed to a minimum length of 35 bases and a minimum quality score of 35 using Trimmomatic v0.22 to remove poor-quality reads and adapter sequences. ABySS v1.3.7 with k values from 20 to 40 was used for *de novo* assembly of pair-end reads and *de novo* consensus sequences generated. The *de novo* consensus sequences were aligned to the consensus sequences deposited in Genbank for each ZIKV using the MUSCLE algorithm in MacVector with Assembler v14.5.3. Results of the comparison of *de novo* and previously deposited sequences are shown in Table S2 for all strains except DakAr41667, for which there was no available consensus sequence until this study (accession MF510857). After comparing and verifying the *de novo* consensus sequences, trimmed pair-end reads were aligned to the respective *de novo* sequence using Bowtie2 v2.2.4 local alignment mode and the very sensitive default setting consisting of Qphred of 20, max number of mismatch of 0, length of seed substring 20, and interval between seed substrings S,1,0.5 to apply a stringent alignment. Following alignment, the sequence alignment map (SAM) was compressed to binary alignment map (BAM) format using Samtool v1.1 and sorted by coordinates with the Picard-tool SortSam v1.128. To limit sequencing biases introduced during the library preparation and sequencing, PCR duplicates were removed from sorted files using Picard-tool MarkDuplicates v1.128, marking reads with an optical duplicate pixel distance of 0 as duplicates. To ensure selected parameters were met prior to downstream analysis, tthe quality and mean read coverage of NGS datasets were determined with Qualimap v2.2. after processing. Sequencing data are available in the ArrayExpress repository (accession E-MTAB-5945). Due to differences in the nucleotide lengths of the genomes across the lineages, SNVs are commonly referred to as the codon number of the protein in which they are located.

***Analysis of data downloaded from the SRA*:** In order to corroborate genetic diversity results in this study with previously reported data, sequence data were downloaded from the Sequence Read Archive (SRA) repository. The SRA contained several ZIKV sequence data entries, including data gathered using the ZIKV strains in the present study. In order to match the SRA dataset to the NGS data in this study, only Illumina platform projects of ZIKV strains in the present analysis were selected for processing and genetic diversity studies. The SRA dataset was analysed using the pipeline in Figure S1, with the exception that the SRA paired-reads were aligned to a reference sequence. The samples chosen were: #1: PA259249, Accession SAMN05789752, Run SRR7879782, aligned to reference KX156775; #2 DakAr41524, Accession SAMN05789756, Run SRR7879856, aligned to reference KX601166; #3 DakAr41524, Accession SAMN05789747, Run SRR7879731, aligned to reference KX198134; #4 MR766_∆E153-156_, Accession SAMN05789757, Run SRR7879861, aligned to reference KX601169; #5 R103451, Accession SAMN05789762, Run SRR7879832, aligned to reference KX694534; #6 DakAr41524, Accession SAMN06711883, Run SRR7879847, aligned to reference KY348860. Because different passage information was given for DakAr41524, several entries were selected for analysis. Each SRA entry for these strains contained 4-10 run files, which were combined for the analysis; thus resulting in one file (no replicates) for each of the samples. It is worth noting that the sequences generated in the present study contained more reads than the entries downloaded from the SRA. Also, in the present study, data from two stocks were analyzed separately, meaning that the reads were not combined during analysis. Overall, the number of mapped reads for the dataset had a range of 24,047-246,285; the coverage had a range of 332-1,611; the mean map quality had a range of 40.0-43.9. All samples were subjected to SNV analysis, with the exception of sample #3 DakAr41524 due to sequence matching and redundance (already have two other DakAr41524 samples). The lower number of reads is possibly due to a difference in propagation or sequencer, as the HiSeq produces more reads than the MiSeq, or the number of samples analysed per sequencing run.

**Supplementary Figure 1**

**
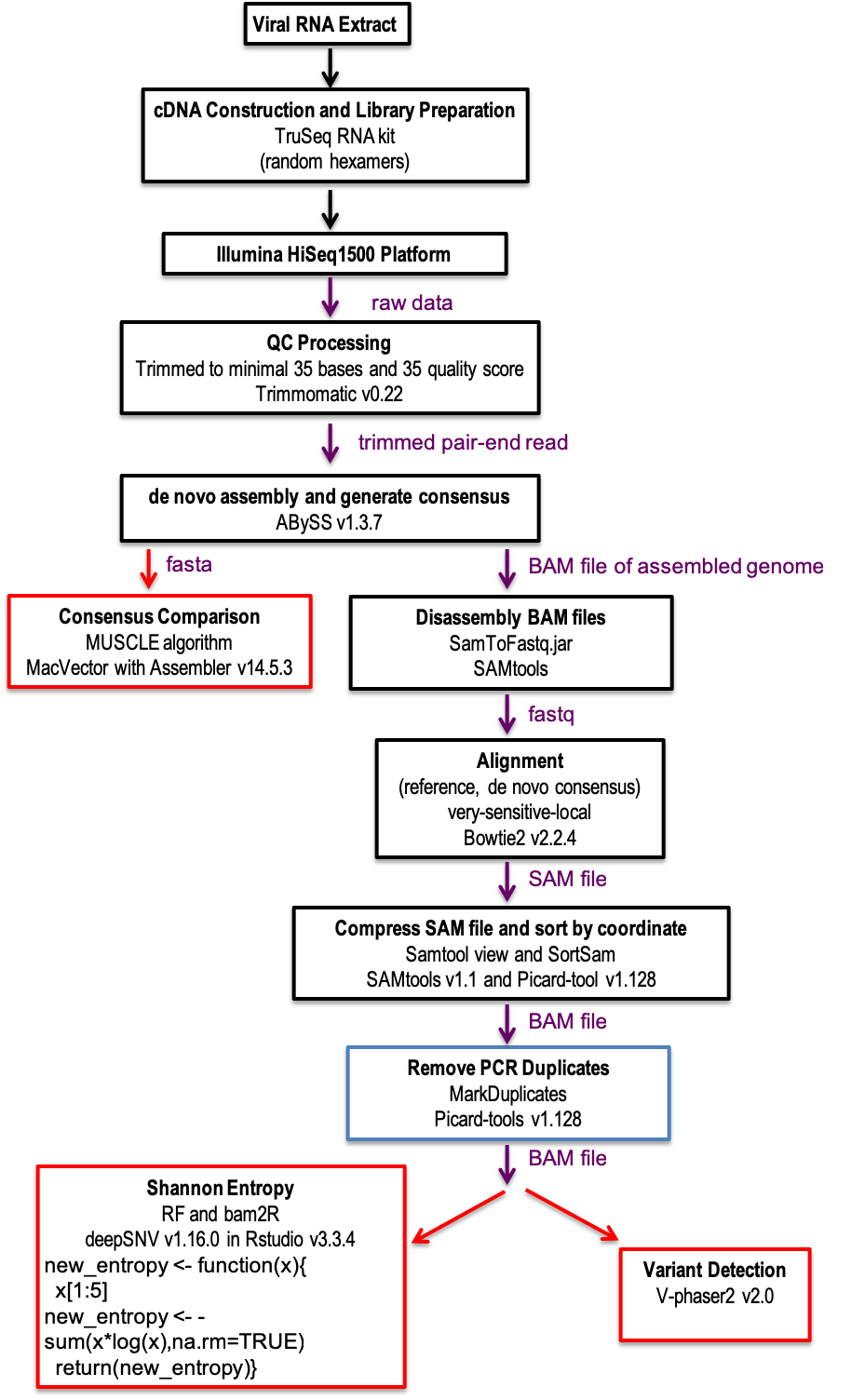
**

**Figure S1. Overview of sequence analysis.** RNA extracted from stock ZIKVs was subjected to NGS using the Illumina HiSeq 1500 platform. Pair-end reads were quality filtered and further processed to reduce sequencing biases prior to performing downstream genetic analysis. Red boxes denote inclusion in results and analyses.

**Supplementary Figure 2**

**Figure S2. Read coverage and mapped reads are highly correlated.** Pearson calculation plot of mean coverage across the genome and the mapped reads shows that the two are highly correlated.

**Supplementary Figure 3**

**Figure S3. Shannon entropy across the genome of each ZIKV.** The mean Shannon entropy (n=2) for each ZIKV at each position was determined and is plotted for nucleotides 107-10,700. To view low and high entropy values, a log-scale was used which does not include entropy values of ‘0’ in the plot. The positions with entropy values greater than two times the SD are shown against a grey background, and the total numbers are provided in Figure 3c.

**Supplementary Figure 4**

**Figure S4. Shannon entropy of each gene region corresponds to gene length.**  Cumulative Shannon entropy calculated for each gene region of each strain (n=2) is depicted as a heat map and corresponds to the gene region length in nucleotides.

**Supplementary Figure 5**

**
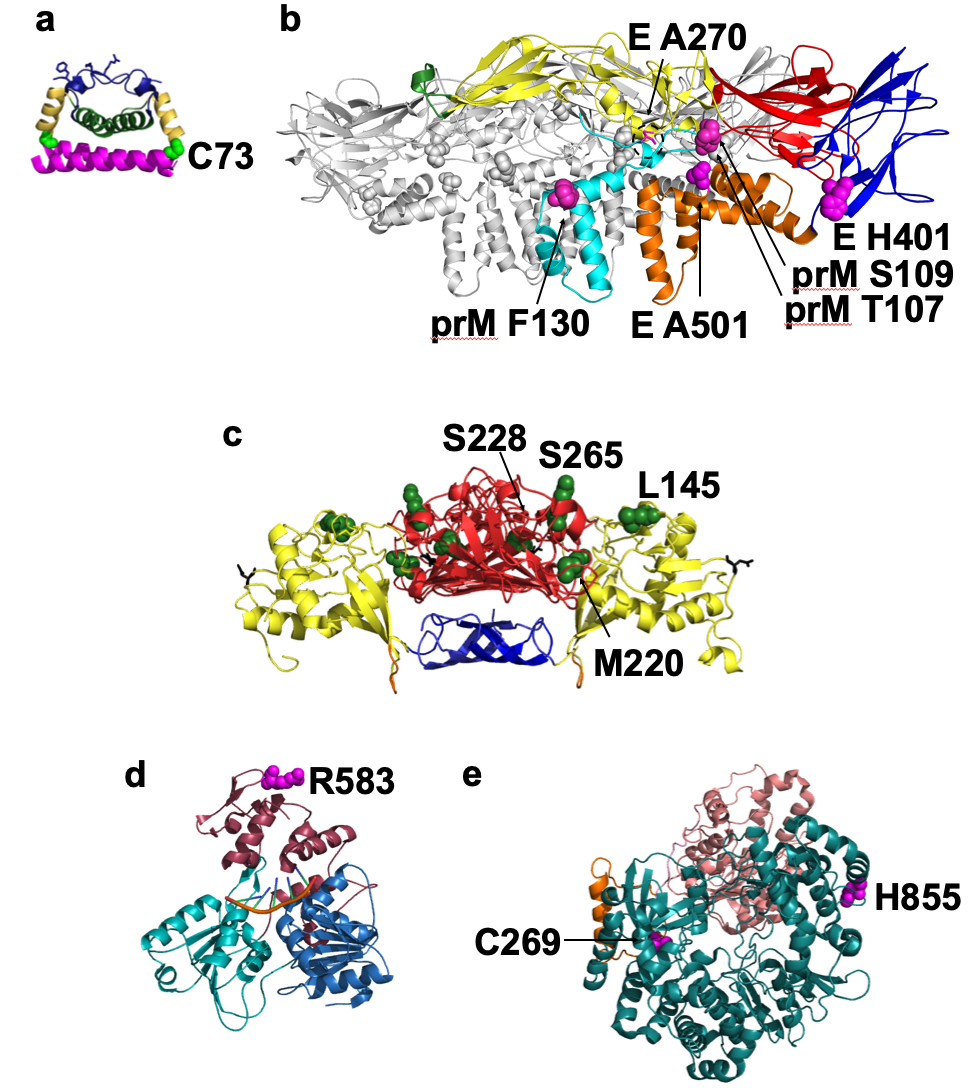
**

**Figure S5. Structural location of shared inter- and intra-lineage SNVs.** Structural rendering of C 73 (green) on the C protein dimer (A). B) SNVs (magenta) in amino acids prM 107, prM109, prM 130, E 270, E 401, and E 501 (magenta) are shown on the side view of the cryo-EM of three M-E proteins; one M and one E protein have been coloured: red - E domain 1, yellow – E domain 2, green- fusion loop, dark blue – E domain 3, orange – E stem and transmembrane domain, cyan – M protein. C) SNVs NS1 145, 220, 228, and 265 (green) rendered on the NS1 protein dimer structure viewed from the side; red: beta-barrel domain, yellow – wing domain, blue - beta-roll, and orange – greasy finger domain. (D) SNV NS3 583 (magenta) shown on the helicase structure; dark blue – domain 1, cyan – domain 2, raspberry – domain 3. E) SNVs NS5 269 and 855 (magenta) depicted on the NS5 protein; pink – methyltransferase, orange – linker, teal – RNA-dependent RNA polymerase. Not shown due to lack of structure availability: NS2A 117, NS4B 86, NS4B 206.

**Supplementary Figure 6**

**
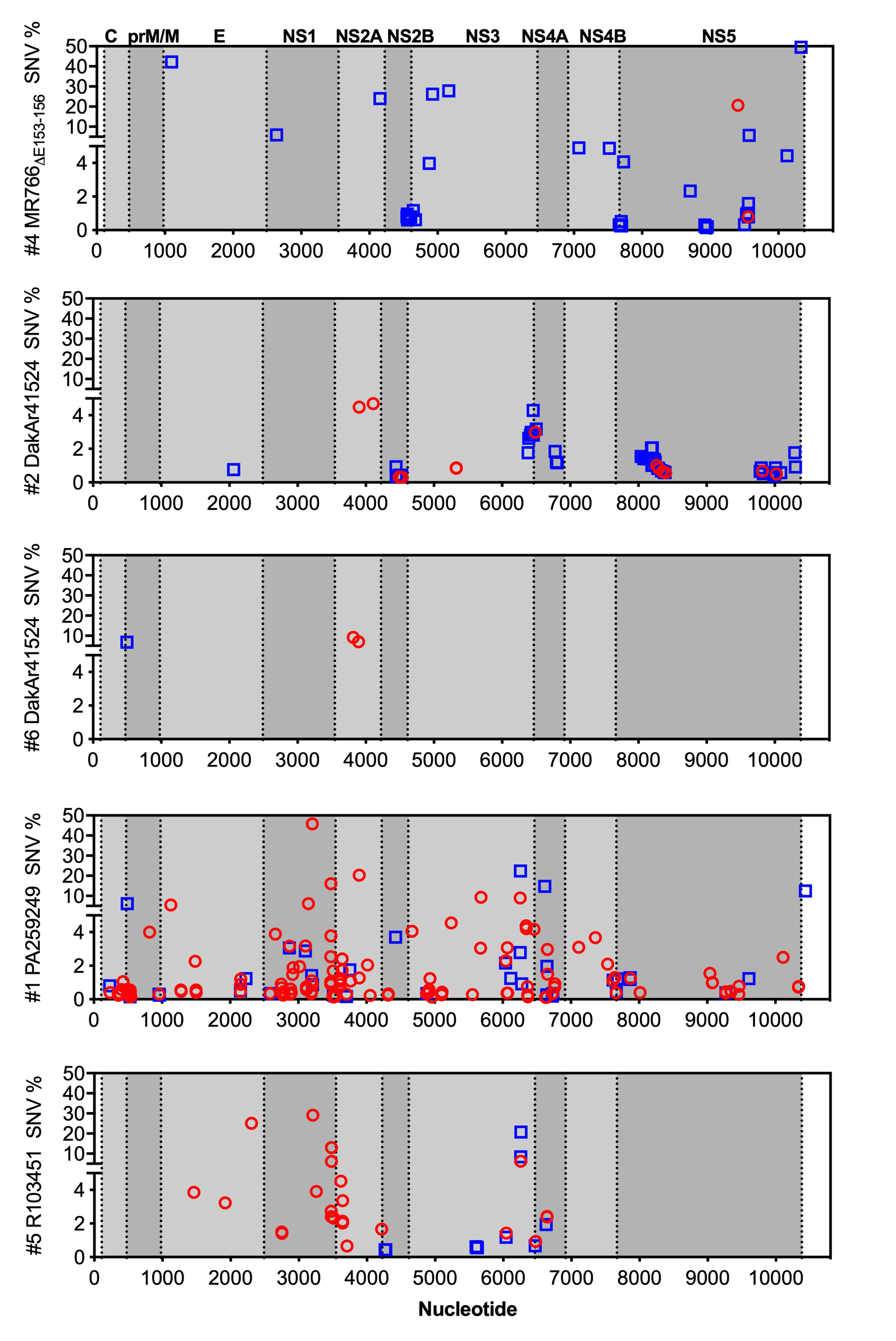
**

**Figure S6. SNV analysis of NGS data downloaded from the SRA database.** A control dataset was downloaded from the SRA database for downstream analysis using the pipeline in this study. Illumina data for ZIKVs was analysed and SNVs were determined using Vphaser2 v2.0. Sample information: PA259249 (sample #1, accession SAMN05789752, run SRR7879782); DakAr41524 (sample #2, accession SAMN05789756, run SRR7879856); MR766_∆E153-156_ (sample #4, accession SAMN05789757, run SRR7879861); R103451 (sample #5, accession SAMN05789762, run SRR7879832); DakAr41524 (sample #6, accession SAMN06711883, run SRR7879847). Red circles denote the frequency and position of non-synonymous SNVs, and blue squares denote the frequency and position of synonymous SNVs.

Supplementary Table 1: Details of the NGS dataset after filtering out poor-quality reads and PCR duplicates.

| Strain | mapped reads* | % of total reads^ | mean coverage (+/- SD) | mean map quality |
| --- | --- | --- | --- | --- |
| MR766 a | 2,088,752 | 32 | 9,586 (+/- 2,199) | 43.93 |
| MR766 b | 2,526,545 | 29 | 10,762 (+/- 2,785) | 43.91 |
| MR766del a | 415,219 | 3 | 1,905 (+/- 572) | 43.87 |
| MR766del b | 830,340 | 5 | 3,523 (+/- 1,134) | 43.87 |
| DakAr41667 a | 1,351,475 | 10 | 6,201 (+/- 1,641) | 43.94 |
| DakAr41667 b | 1,786,439 | 11 | 8,203 (+/- 1,897) | 43.94 |
| DakAr41524 a | 1,655,915 | 13 | 7,598 (+/- 1,939) | 43.95 |
| DakAr41524 b | 2,001,443 | 15 | 9,189 (+/- 2,125) | 43.94 |
| FSS13025 a | 1,518,968 | 20 | 6,966 (+/- 2,052) | 43.93 |
| FSS13025 b | 2,200,874 | 13 | 10,116 (+/- 2,470) | 43.93 |
| PA259249 a | 1,609,562 | 12 | 7,377 (+/- 1,950) | 43.94 |
| PA259249 b | 1,178,313 | 10 | 5,407 (+/- 1,390) | 43.93 |
| R103451 a | 1,806,499 | 20 | 8,287 (+/- 1,965) | 43.93 |
| R103451 b | 1,850,495 | 15 | 8,492 (+/- 2,053) | 43.95 |

*The reads that mapped to the ZIKV sequence are listed twice: as number of reads that mapped* or as a percentage of the total number of reads^.

Supplementary Table 2: Comparison of *de novo* sequences to publicly available genome sequences.

| Strain | Accession | Nucleotide difference | Amino Acid difference |
| --- | --- | --- | --- |
| MR766 | KX830960 | 5177 (C to T), 10774 (T to A), 10775 (T to A) | None |
| MR766_∆E153-156_ | KU955594 | None | None |
| DakAr41524 | KX601166 | None | None |
| FSS13025 | KU955593 | 3282 (G to A) | 1059 (K to E) |
| PA259249 | KX156775 | 56 (A to G) | None |
| R103451 | KX694534 | 2305 (G to A) | 733 (R to K) |

Supplementary Table 3: Gene regions of each ZIKV strain.

|  | MR766 DakAr41667 DakAr41524 | MR766_∆E153-156_ | FSS13025 PA259249 R103451 |
| --- | --- | --- | --- |
| 5'UTR | 1-106 | 1-106 | 1-107 |
| C | 107-472 | 107-472 | 108-473 |
| prM/M | 473-976 | 473-976 | 474-977 |
| E | 977-2488 | 977-2476 | 978-2489 |
| NS1 | 2489-3544 | 2477-3532 | 2490-3545 |
| NS2A | 3545-4222 | 3533-4210 | 3546-4223 |
| NS2B | 4223-4612 | 4211-4600 | 4224-4613 |
| NS3 | 4613-6463 | 4601-6451 | 4614-6464 |
| NS4A | 6464-6913 | 6452-6901 | 6465-6914 |
| NS4B | 6914-7666 | 6902-7654 | 6915-7667 |
| NS5 | 7667-10378 | 7655-10366 | 7668-10379 |
| 3'UTR | 10379-10795 | 10367-10794 | 10380-10802 |

Supplementary Table 4: Complete list of synonymous SNVs.

| ZIKV | Replicate | Position | Consensus | Variant | % |
| --- | --- | --- | --- | --- | --- |
| MR766 | a | 178 | A | G | 0.425 |
| MR766 | b | 178 | A | G | 0.252 |
| MR766 | a | 208 | A | G | 0.199 |
| MR766 | a | 325 | G | A | 0.537 |
| MR766 | b | 325 | G | A | 0.447 |
| MR766 | a | 400 | G | A | 0.038 |
| MR766 | a | 682 | C | T | 0.421 |
| MR766 | b | 682 | C | T | 0.380 |
| MR766 | a | 709 | A | G | 0.287 |
| MR766 | b | 709 | A | G | 0.355 |
| MR766 | a | 754 | C | T | 0.320 |
| MR766 | b | 754 | C | T | 0.597 |
| MR766 | a | 781 | G | A | 0.475 |
| MR766 | b | 799 | G | A | 0.389 |
| MR766 | b | 889 | C | A | 0.498 |
| MR766 | a | 908 | T | C | 0.296 |
| MR766 | b | 908 | T | C | 0.311 |
| MR766 | b | 955 | G | T | 0.225 |
| MR766 | a | 1099 | T | C | 2.204 |
| MR766 | b | 1099 | T | C | 2.613 |
| MR766 | a | 1246 | T | C | 0.191 |
| MR766 | a | 1366 | T | C | 0.325 |
| MR766 | b | 1586 | C | T | 0.182 |
| MR766 | a | 1666 | C | T | 0.167 |
| MR766 | b | 1666 | C | T | 0.163 |
| MR766 | a | 1774 | C | T | 0.370 |
| MR766 | b | 1774 | C | T | 0.454 |
| MR766 | b | 1804 | G | A | 0.694 |
| MR766 | b | 1894 | C | T | 0.127 |
| MR766 | a | 2080 | C | T | 0.597 |
| MR766 | b | 2080 | C | T | 0.663 |
| MR766 | a | 2164 | C | T | 0.712 |
| MR766 | a | 2200 | A | G | 0.606 |
| MR766 | b | 2200 | A | G | 0.493 |
| MR766 | a | 2440 | C | T | 0.694 |
| MR766 | b | 2440 | C | T | 0.802 |
| MR766 | a | 2503 | A | G | 0.188 |
| MR766 | b | 2503 | A | G | 0.087 |
| MR766 | b | 2536 | C | T | 0.403 |
| MR766 | a | 2608 | C | T | 0.337 |
| MR766 | b | 2608 | C | T | 0.213 |
| MR766 | a | 2923 | T | C | 0.297 |
| MR766 | b | 2923 | T | C | 0.287 |
| MR766 | a | 3064 | G | A | 15.370 |
| MR766 | b | 3064 | G | A | 19.900 |
| MR766 | a | 3082 | G | T | 0.172 |
| MR766 | b | 3082 | G | T | 0.148 |
| MR766 | a | 3145 | G | A | 0.057 |
| MR766 | a | 3175 | C | T | 0.219 |
| MR766 | b | 3175 | C | T | 0.179 |
| MR766 | a | 3253 | C | T | 0.559 |
| MR766 | a | 3589 | G | A | 0.239 |
| MR766 | a | 3589 | G | T | 0.168 |
| MR766 | b | 3589 | G | T | 0.166 |
| MR766 | b | 3793 | T | C | 0.222 |
| MR766 | b | 3808 | C | A | 0.078 |
| MR766 | a | 3931 | T | C | 0.291 |
| MR766 | a | 3994 | C | T | 0.723 |
| MR766 | b | 3994 | C | T | 0.644 |
| MR766 | a | 4039 | A | G | 0.047 |
| MR766 | a | 4051 | A | G | 0.062 |
| MR766 | a | 4057 | A | G | 0.300 |
| MR766 | b | 4150 | A | T | 0.221 |
| MR766 | b | 4168 | A | G | 11.250 |
| MR766 | a | 4180 | T | C | 4.560 |
| MR766 | b | 4180 | T | C | 4.953 |
| MR766 | b | 4213 | T | C | 0.192 |
| MR766 | b | 4321 | T | C | 0.221 |
| MR766 | b | 4360 | A | G | 0.352 |
| MR766 | a | 4372 | C | T | 0.348 |
| MR766 | b | 4372 | C | T | 0.173 |
| MR766 | a | 4454 | C | T | 0.185 |
| MR766 | b | 4454 | C | T | 0.203 |
| MR766 | a | 4684 | A | G | 0.203 |
| MR766 | a | 4747 | C | T | 0.518 |
| MR766 | b | 4747 | C | T | 0.443 |
| MR766 | a | 4828 | C | T | 0.397 |
| MR766 | b | 4828 | C | T | 0.375 |
| MR766 | a | 4873 | A | G | 0.046 |
| MR766 | a | 4891 | C | T | 6.201 |
| MR766 | b | 4891 | C | T | 7.716 |
| MR766 | a | 4939 | T | C | 20.180 |
| MR766 | b | 4939 | T | C | 14.200 |
| MR766 | a | 5029 | C | A | 0.438 |
| MR766 | b | 5029 | C | A | 0.636 |
| MR766 | a | 5080 | C | A | 0.043 |
| MR766 | b | 5080 | C | T | 0.111 |
| MR766 | b | 5116 | A | G | 0.212 |
| MR766 | a | 5206 | C | A | 0.054 |
| MR766 | a | 5296 | C | T | 0.304 |
| MR766 | b | 5296 | C | T | 0.181 |
| MR766 | b | 5341 | C | T | 0.125 |
| MR766 | a | 5416 | A | C | 1.594 |
| MR766 | b | 5416 | A | C | 1.586 |
| MR766 | a | 5455 | C | T | 0.190 |
| MR766 | b | 5455 | C | T | 0.267 |
| MR766 | a | 5599 | T | C | 0.242 |
| MR766 | a | 5611 | C | T | 0.557 |
| MR766 | b | 5611 | C | T | 0.449 |
| MR766 | a | 5650 | C | T | 0.377 |
| MR766 | b | 5650 | C | T | 0.377 |
| MR766 | a | 5746 | G | A | 1.363 |
| MR766 | b | 5746 | G | A | 1.127 |
| MR766 | a | 5830 | C | T | 0.458 |
| MR766 | b | 5830 | C | T | 0.459 |
| MR766 | a | 5845 | C | T | 1.027 |
| MR766 | b | 5845 | C | T | 0.990 |
| MR766 | a | 5947 | C | T | 0.353 |
| MR766 | b | 5947 | C | T | 0.301 |
| MR766 | a | 6136 | T | C | 0.149 |
| MR766 | a | 6790 | C | T | 0.335 |
| MR766 | a | 6877 | C | T | 0.214 |
| MR766 | b | 6877 | C | T | 0.348 |
| MR766 | a | 6959 | C | T | 0.568 |
| MR766 | b | 6959 | C | T | 0.465 |
| MR766 | a | 7036 | T | A | 0.259 |
| MR766 | b | 7036 | T | A | 0.308 |
| MR766 | a | 7084 | G | A | 0.353 |
| MR766 | a | 7090 | C | T | 0.443 |
| MR766 | b | 7090 | C | T | 0.359 |
| MR766 | a | 7102 | C | T | 0.177 |
| MR766 | b | 7102 | C | T | 0.150 |
| MR766 | a | 7501 | A | G | 0.107 |
| MR766 | a | 7531 | G | A | 0.402 |
| MR766 | b | 7531 | G | A | 0.547 |
| MR766 | a | 7627 | C | T | 0.780 |
| MR766 | b | 7627 | C | T | 0.737 |
| MR766 | a | 7951 | C | T | 0.454 |
| MR766 | a | 8014 | G | C | 0.358 |
| MR766 | b | 8014 | G | C | 0.253 |
| MR766 | a | 8137 | A | G | 0.227 |
| MR766 | b | 8164 | T | C | 0.090 |
| MR766 | b | 8314 | A | G | 0.252 |
| MR766 | a | 8392 | A | G | 0.082 |
| MR766 | a | 8404 | C | T | 0.096 |
| MR766 | a | 8719 | C | T | 5.528 |
| MR766 | b | 8719 | C | T | 3.775 |
| MR766 | a | 8749 | C | T | 0.188 |
| MR766 | b | 8773 | A | G | 0.215 |
| MR766 | a | 8810 | C | T | 0.715 |
| MR766 | b | 8810 | C | T | 0.648 |
| MR766 | a | 8854 | C | A | 0.442 |
| MR766 | b | 8854 | C | A | 0.384 |
| MR766 | a | 8977 | T | C | 0.274 |
| MR766 | b | 8977 | T | C | 0.317 |
| MR766 | a | 9382 | C | T | 0.424 |
| MR766 | b | 9382 | C | T | 0.704 |
| MR766 | a | 9581 | T | C | 6.837 |
| MR766 | b | 9581 | T | C | 6.755 |
| MR766 | a | 9664 | C | T | 0.453 |
| MR766 | b | 9664 | C | T | 0.446 |
| MR766 | a | 9688 | T | C | 0.528 |
| MR766 | a | 9901 | G | A | 1.259 |
| MR766 | b | 9901 | G | A | 1.150 |
| MR766 | a | 10072 | G | A | 0.343 |
| MR766 | b | 10072 | G | A | 0.638 |
| MR766 | a | 10138 | C | T | 0.548 |
| MR766 | b | 10138 | C | T | 0.506 |
| MR766 | a | 10207 | A | G | 0.413 |
| MR766 | b | 10207 | A | G | 0.302 |
| MR766 | a | 10258 | C | T | 0.169 |
| MR766 | b | 10285 | C | T | 0.498 |
| MR766 | b | 10312 | C | T | 0.161 |
| MR766 | a | 10476 | C | T | 0.410 |
| MR766 | b | 10476 | C | T | 0.353 |
| MR766 | a | 10490 | G | T | 0.781 |
| MR766 | b | 10490 | G | T | 0.786 |
| MR766_∆E153-156_ | a | 325 | G | A | 0.607 |
| MR766_∆E153-156_ | b | 325 | G | A | 0.262 |
| MR766_∆E153-156_ | a | 454 | G | C | 0.521 |
| MR766_∆E153-156_ | a | 785 | T | C | 0.511 |
| MR766_∆E153-156_ | a | 874 | G | A | 1.172 |
| MR766_∆E153-156_ | b | 1018 | C | T | 0.326 |
| MR766_∆E153-156_ | a | 1156 | C | T | 1.151 |
| MR766_∆E153-156_ | b | 1156 | C | T | 0.571 |
| MR766_∆E153-156_ | a | 1387 | T | C | 2.272 |
| MR766_∆E153-156_ | b | 1387 | T | C | 3.836 |
| MR766_∆E153-156_ | a | 1399 | A | T | 0.444 |
| MR766_∆E153-156_ | b | 1399 | A | T | 0.478 |
| MR766_∆E153-156_ | a | 1411 | C | T | 1.750 |
| MR766_∆E153-156_ | b | 1411 | C | T | 1.075 |
| MR766_∆E153-156_ | a | 1477 | T | C | 2.508 |
| MR766_∆E153-156_ | a | 1645 | T | C | 0.416 |
| MR766_∆E153-156_ | a | 1729 | C | T | 9.214 |
| MR766_∆E153-156_ | b | 1729 | C | T | 3.093 |
| MR766_∆E153-156_ | a | 1870 | C | T | 0.565 |
| MR766_∆E153-156_ | b | 1870 | C | T | 0.346 |
| MR766_∆E153-156_ | a | 2008 | C | T | 0.692 |
| MR766_∆E153-156_ | b | 2008 | C | T | 0.275 |
| MR766_∆E153-156_ | a | 2455 | C | T | 0.442 |
| MR766_∆E153-156_ | a | 2473 | T | C | 3.070 |
| MR766_∆E153-156_ | b | 2473 | T | C | 2.999 |
| MR766_∆E153-156_ | a | 2491 | A | G | 0.362 |
| MR766_∆E153-156_ | b | 2836 | A | G | 0.355 |
| MR766_∆E153-156_ | a | 2848 | T | C | 0.861 |
| MR766_∆E153-156_ | a | 2893 | A | G | 0.550 |
| MR766_∆E153-156_ | a | 3100 | C | T | 0.699 |
| MR766_∆E153-156_ | a | 3271 | A | G | 0.932 |
| MR766_∆E153-156_ | b | 3271 | A | G | 0.649 |
| MR766_∆E153-156_ | b | 3283 | C | T | 0.913 |
| MR766_∆E153-156_ | a | 3460 | C | T | 1.211 |
| MR766_∆E153-156_ | a | 3692 | C | T | 0.644 |
| MR766_∆E153-156_ | b | 4934 | C | T | 0.399 |
| MR766_∆E153-156_ | a | 5152 | G | A | 0.980 |
| MR766_∆E153-156_ | b | 5191 | A | G | 0.510 |
| MR766_∆E153-156_ | a | 5791 | A | G | 0.478 |
| MR766_∆E153-156_ | b | 5791 | A | G | 0.923 |
| MR766_∆E153-156_ | b | 6170 | C | T | 0.202 |
| MR766_∆E153-156_ | a | 6412 | G | T | 0.761 |
| MR766_∆E153-156_ | b | 6427 | C | T | 0.199 |
| MR766_∆E153-156_ | a | 6466 | A | G | 0.163 |
| MR766_∆E153-156_ | b | 6958 | G | A | 0.141 |
| MR766_∆E153-156_ | a | 7072 | G | A | 9.691 |
| MR766_∆E153-156_ | b | 7072 | G | A | 11.810 |
| MR766_∆E153-156_ | a | 7087 | C | T | 0.643 |
| MR766_∆E153-156_ | a | 7156 | A | G | 1.586 |
| MR766_∆E153-156_ | a | 7519 | G | A | 9.732 |
| MR766_∆E153-156_ | b | 7519 | G | A | 12.470 |
| MR766_∆E153-156_ | b | 7582 | C | T | 0.523 |
| MR766_∆E153-156_ | b | 7678 | G | A | 0.298 |
| MR766_∆E153-156_ | b | 7729 | C | T | 1.657 |
| MR766_∆E153-156_ | b | 7741 | G | A | 0.413 |
| MR766_∆E153-156_ | a | 7804 | A | G | 0.415 |
| MR766_∆E153-156_ | a | 7819 | A | G | 0.707 |
| MR766_∆E153-156_ | a | 7888 | T | C | 0.576 |
| MR766_∆E153-156_ | b | 8512 | C | T | 0.220 |
| MR766_∆E153-156_ | a | 8545 | A | G | 0.995 |
| MR766_∆E153-156_ | b | 8545 | A | G | 1.728 |
| MR766_∆E153-156_ | b | 8590 | C | T | 0.098 |
| MR766_∆E153-156_ | b | 8833 | C | T | 1.763 |
| MR766_∆E153-156_ | a | 8845 | A | G | 0.484 |
| MR766_∆E153-156_ | b | 8845 | A | G | 0.682 |
| MR766_∆E153-156_ | a | 8986 | G | T | 2.238 |
| MR766_∆E153-156_ | b | 8986 | G | T | 0.232 |
| MR766_∆E153-156_ | a | 9085 | C | T | 0.436 |
| MR766_∆E153-156_ | b | 9085 | C | T | 0.402 |
| MR766_∆E153-156_ | a | 9310 | T | C | 0.616 |
| MR766_∆E153-156_ | b | 9757 | A | T | 0.100 |
| MR766_∆E153-156_ | a | 9877 | C | T | 0.773 |
| MR766_∆E153-156_ | a | 10081 | G | A | 0.986 |
| MR766_∆E153-156_ | a | 10377 | T | C | 2.283 |
| MR766_∆E153-156_ | b | 10377 | T | C | 2.520 |
| MR766_∆E153-156_ | a | 10432 | A | G | 1.392 |
| MR766_∆E153-156_ | a | 10501 | C | T | 0.719 |
| MR766_∆E153-156_ | b | 10501 | C | T | 0.676 |
| MR766_∆E153-156_ | a | 10536 | C | T | 1.000 |
| DakAr41667 | b | 1313 | T | C | 0.063 |
| DakAr41667 | b | 1405 | G | T | 0.065 |
| DakAr41667 | b | 1459 | C | T | 0.037 |
| DakAr41667 | b | 1462 | A | G | 0.027 |
| DakAr41667 | b | 1465 | C | T | 0.065 |
| DakAr41667 | b | 1471 | A | G | 0.053 |
| DakAr41667 | a | 2767 | A | G | 0.330 |
| DakAr41667 | b | 2767 | A | G | 0.394 |
| DakAr41667 | b | 3274 | T | C | 0.057 |
| DakAr41667 | b | 3391 | T | C | 0.260 |
| DakAr41667 | a | 3568 | C | T | 0.189 |
| DakAr41667 | b | 3568 | C | T | 0.240 |
| DakAr41667 | a | 3613 | T | C | 0.576 |
| DakAr41667 | b | 3613 | T | C | 0.624 |
| DakAr41667 | a | 4567 | C | T | 10.030 |
| DakAr41667 | b | 4567 | C | T | 9.560 |
| DakAr41667 | a | 4753 | C | T | 10.300 |
| DakAr41667 | b | 4753 | C | T | 10.140 |
| DakAr41667 | a | 5629 | A | G | 0.702 |
| DakAr41667 | b | 5629 | A | G | 0.732 |
| DakAr41667 | b | 5887 | C | T | 38.510 |
| DakAr41667 | b | 5908 | A | G | 0.051 |
| DakAr41667 | b | 6037 | T | C | 0.311 |
| DakAr41667 | b | 6091 | T | C | 0.071 |
| DakAr41667 | b | 6100 | C | T | 0.110 |
| DakAr41667 | b | 6154 | G | A | 0.030 |
| DakAr41667 | b | 6223 | G | A | 0.069 |
| DakAr41667 | b | 6232 | C | T | 0.053 |
| DakAr41667 | b | 6238 | C | T | 0.066 |
| DakAr41667 | b | 6310 | T | G | 0.042 |
| DakAr41667 | b | 7036 | T | C | 2.911 |
| DakAr41667 | a | 7174 | T | C | 0.407 |
| DakAr41667 | b | 7174 | T | C | 0.504 |
| DakAr41667 | b | 7348 | A | G | 0.066 |
| DakAr41667 | b | 7351 | T | C | 0.076 |
| DakAr41667 | b | 7354 | C | T | 0.092 |
| DakAr41667 | b | 7555 | C | T | 0.158 |
| DakAr41667 | a | 7630 | C | T | 0.177 |
| DakAr41667 | b | 7669 | A | G | 0.057 |
| DakAr41667 | b | 8038 | T | C | 0.267 |
| DakAr41667 | a | 8341 | C | T | 0.225 |
| DakAr41667 | b | 8341 | C | T | 0.222 |
| DakAr41667 | a | 8557 | A | G | 0.248 |
| DakAr41667 | b | 8890 | A | G | 0.150 |
| DakAr41667 | a | 9790 | T | C | 39.860 |
| DakAr41667 | b | 10192 | G | A | 0.049 |
| DakAr41667 | a | 10592 | T | C | 0.475 |
| DakAr41524 | b | 853 | T | C | 0.194 |
| DakAr41524 | a | 3109 | T | C | 0.088 |
| DakAr41524 | a | 4537 | C | T | 0.214 |
| DakAr41524 | b | 4537 | C | T | 0.184 |
| DakAr41524 | a | 4636 | T | A | 0.157 |
| DakAr41524 | a | 4858 | T | G | 0.251 |
| DakAr41524 | b | 4858 | T | G | 0.537 |
| DakAr41524 | a | 5185 | C | T | 0.235 |
| DakAr41524 | b | 5185 | C | T | 0.254 |
| DakAr41524 | a | 5287 | C | T | 0.687 |
| DakAr41524 | b | 5287 | C | T | 0.460 |
| DakAr41524 | a | 5491 | A | T | 0.220 |
| DakAr41524 | b | 5491 | A | T | 0.264 |
| DakAr41524 | a | 5936 | C | T | 20.080 |
| DakAr41524 | b | 6376 | A | G | 0.063 |
| DakAr41524 | b | 9352 | C | T | 0.190 |
| DakAr41524 | a | 9538 | C | T | 1.278 |
| DakAr41524 | b | 9673 | A | G | 8.357 |
| DakAr41524 | a | 10228 | G | A | 0.076 |
| DakAr41524 | b | 10228 | G | T | 0.272 |
| DakAr41524 | a | 10410 | T | C | 0.248 |
| FSS13025 | b | 140 | C | T | 0.288 |
| FSS13025 | b | 267 | T | C | 0.217 |
| FSS13025 | a | 326 | G | A | 0.470 |
| FSS13025 | b | 326 | G | A | 0.269 |
| FSS13025 | b | 431 | T | C | 0.276 |
| FSS13025 | a | 464 | A | T | 0.405 |
| FSS13025 | b | 464 | A | T | 0.175 |
| FSS13025 | a | 473 | A | T | 0.341 |
| FSS13025 | b | 473 | A | T | 0.183 |
| FSS13025 | b | 569 | T | C | 0.570 |
| FSS13025 | b | 602 | C | T | 0.208 |
| FSS13025 | a | 686 | G | C | 0.045 |
| FSS13025 | a | 794 | G | A | 1.114 |
| FSS13025 | b | 794 | G | A | 1.373 |
| FSS13025 | b | 926 | C | T | 0.225 |
| FSS13025 | a | 986 | C | T | 0.295 |
| FSS13025 | b | 986 | C | T | 0.207 |
| FSS13025 | b | 1133 | C | T | 0.045 |
| FSS13025 | a | 1208 | A | G | 0.383 |
| FSS13025 | b | 1208 | A | G | 0.327 |
| FSS13025 | b | 1238 | C | T | 0.085 |
| FSS13025 | a | 1349 | A | G | 3.845 |
| FSS13025 | b | 1349 | A | G | 4.309 |
| FSS13025 | a | 1442 | T | C | 1.131 |
| FSS13025 | b | 1442 | T | C | 1.060 |
| FSS13025 | a | 1742 | C | T | 0.456 |
| FSS13025 | b | 1841 | C | T | 0.914 |
| FSS13025 | a | 1856 | G | A | 0.361 |
| FSS13025 | b | 1856 | G | A | 0.908 |
| FSS13025 | a | 1886 | G | T | 0.603 |
| FSS13025 | b | 1886 | G | T | 0.477 |
| FSS13025 | a | 1892 | C | T | 0.976 |
| FSS13025 | b | 1892 | C | T | 0.884 |
| FSS13025 | b | 2042 | T | C | 0.164 |
| FSS13025 | b | 2192 | C | T | 0.305 |
| FSS13025 | a | 2552 | C | T | 0.225 |
| FSS13025 | b | 2552 | C | T | 0.209 |
| FSS13025 | a | 2627 | C | T | 0.418 |
| FSS13025 | b | 2627 | C | T | 0.679 |
| FSS13025 | b | 2633 | A | G | 0.176 |
| FSS13025 | b | 2882 | C | T | 0.172 |
| FSS13025 | b | 3176 | C | T | 0.161 |
| FSS13025 | a | 3269 | C | T | 0.176 |
| FSS13025 | a | 3594 | C | T | 0.189 |
| FSS13025 | a | 3704 | C | T | 0.421 |
| FSS13025 | b | 3704 | C | T | 0.530 |
| FSS13025 | a | 4106 | G | A | 0.053 |
| FSS13025 | a | 4160 | T | C | 0.444 |
| FSS13025 | b | 4160 | T | C | 0.500 |
| FSS13025 | b | 4175 | C | T | 0.233 |
| FSS13025 | a | 4190 | G | A | 0.780 |
| FSS13025 | b | 4190 | G | A | 1.507 |
| FSS13025 | b | 4427 | T | C | 0.197 |
| FSS13025 | b | 4445 | C | T | 0.344 |
| FSS13025 | a | 4580 | A | G | 0.234 |
| FSS13025 | a | 4679 | G | C | 0.197 |
| FSS13025 | a | 4733 | C | T | 0.873 |
| FSS13025 | a | 5036 | T | C | 0.434 |
| FSS13025 | b | 5036 | T | C | 0.517 |
| FSS13025 | a | 5163 | C | T | 1.259 |
| FSS13025 | b | 5163 | C | T | 3.996 |
| FSS13025 | b | 5306 | A | G | 0.217 |
| FSS13025 | b | 5339 | T | C | 0.240 |
| FSS13025 | a | 5813 | T | C | 0.260 |
| FSS13025 | b | 5813 | T | C | 0.330 |
| FSS13025 | b | 5906 | G | A | 0.070 |
| FSS13025 | a | 5912 | C | T | 0.342 |
| FSS13025 | b | 5912 | C | T | 0.539 |
| FSS13025 | a | 6362 | A | G | 0.256 |
| FSS13025 | b | 6362 | A | G | 0.752 |
| FSS13025 | b | 6677 | C | T | 0.067 |
| FSS13025 | a | 6857 | C | T | 0.736 |
| FSS13025 | b | 6857 | C | T | 1.502 |
| FSS13025 | b | 6989 | T | C | 32.330 |
| FSS13025 | a | 7172 | C | T | 0.361 |
| FSS13025 | b | 7172 | C | T | 0.246 |
| FSS13025 | a | 7239 | C | T | 1.849 |
| FSS13025 | b | 7239 | C | T | 2.807 |
| FSS13025 | b | 7556 | C | T | 0.172 |
| FSS13025 | a | 7571 | A | G | 0.269 |
| FSS13025 | a | 7592 | T | C | 2.097 |
| FSS13025 | b | 7592 | T | C | 2.269 |
| FSS13025 | a | 7653 | T | C | 0.204 |
| FSS13025 | a | 7667 | T | C | 22.270 |
| FSS13025 | b | 7763 | C | T | 0.227 |
| FSS13025 | b | 7820 | A | G | 0.445 |
| FSS13025 | b | 7988 | C | T | 6.730 |
| FSS13025 | b | 8273 | T | C | 0.514 |
| FSS13025 | b | 8276 | G | A | 0.282 |
| FSS13025 | b | 9038 | A | G | 0.308 |
| FSS13025 | b | 9041 | A | G | 0.118 |
| FSS13025 | a | 9272 | T | C | 1.687 |
| FSS13025 | b | 9350 | G | A | 0.208 |
| FSS13025 | b | 9545 | G | A | 0.036 |
| FSS13025 | b | 9932 | C | T | 0.086 |
| FSS13025 | b | 10232 | C | T | 0.251 |
| FSS13025 | a | 10391 | A | G | 0.382 |
| FSS13025 | a | 10528 | G | C | 0.087 |
| FSS13025 | a | 10612 | T | C | 0.289 |
| PA259249 | a | 326 | G | A | 0.282 |
| PA259249 | a | 491 | C | T | 7.473 |
| PA259249 | b | 491 | C | T | 3.191 |
| PA259249 | b | 639 | C | T | 0.236 |
| PA259249 | a | 1127 | C | T | 0.223 |
| PA259249 | b | 1940 | A | G | 0.474 |
| PA259249 | a | 2663 | C | T | 0.253 |
| PA259249 | a | 2936 | A | G | 0.424 |
| PA259249 | b | 2936 | A | G | 0.416 |
| PA259249 | a | 3914 | C | T | 0.620 |
| PA259249 | b | 3992 | C | T | 0.259 |
| PA259249 | a | 4181 | C | T | 0.235 |
| PA259249 | b | 4184 | C | T | 5.414 |
| PA259249 | b | 5120 | G | A | 0.367 |
| PA259249 | a | 5285 | A | T | 0.196 |
| PA259249 | b | 5597 | C | T | 0.187 |
| PA259249 | a | 5861 | C | T | 0.336 |
| PA259249 | a | 5901 | C | T | 0.248 |
| PA259249 | b | 6008 | T | C | 0.557 |
| PA259249 | b | 6827 | T | C | 0.277 |
| PA259249 | a | 6845 | A | G | 0.191 |
| PA259249 | b | 7250 | C | T | 0.394 |
| PA259249 | a | 7646 | C | T | 0.464 |
| PA259249 | b | 7646 | C | T | 0.444 |
| PA259249 | a | 8051 | T | C | 0.298 |
| PA259249 | b | 8051 | T | C | 0.469 |
| PA259249 | a | 8204 | T | C | 1.421 |
| PA259249 | b | 8204 | T | C | 1.379 |
| PA259249 | a | 8234 | C | T | 0.059 |
| PA259249 | a | 8267 | T | C | 0.085 |
| PA259249 | b | 9488 | C | T | 0.290 |
| PA259249 | a | 10055 | C | T | 0.198 |
| PA259249 | a | 10268 | C | T | 0.467 |
| PA259249 | b | 10268 | C | T | 0.558 |
| R103451 | b | 248 | C | T | 0.066 |
| R103451 | b | 257 | T | A | 0.062 |
| R103451 | b | 266 | T | C | 0.066 |
| R103451 | b | 278 | G | A | 0.090 |
| R103451 | a | 326 | G | A | 0.439 |
| R103451 | b | 326 | G | A | 0.529 |
| R103451 | a | 1364 | C | T | 0.370 |
| R103451 | b | 1364 | C | T | 0.378 |
| R103451 | b | 1466 | T | C | 0.305 |
| R103451 | a | 1787 | T | C | 0.541 |
| R103451 | b | 1787 | T | C | 0.467 |
| R103451 | a | 1799 | G | A | 0.071 |
| R103451 | a | 1973 | C | T | 1.852 |
| R103451 | b | 1973 | C | T | 1.400 |
| R103451 | b | 2537 | T | C | 0.280 |
| R103451 | a | 2714 | C | T | 0.147 |
| R103451 | b | 2951 | T | C | 0.049 |
| R103451 | b | 3269 | C | T | 0.133 |
| R103451 | b | 4433 | C | T | 0.151 |
| R103451 | b | 4479 | C | T | 0.057 |
| R103451 | b | 4484 | G | A | 0.073 |
| R103451 | a | 4709 | T | C | 0.269 |
| R103451 | b | 4982 | T | C | 0.276 |
| R103451 | b | 5117 | G | A | 0.159 |
| R103451 | b | 5123 | G | A | 0.101 |
| R103451 | a | 5357 | C | T | 0.308 |
| R103451 | b | 5357 | C | T | 0.379 |
| R103451 | b | 5774 | C | T | 0.223 |
| R103451 | a | 7619 | T | C | 3.373 |
| R103451 | b | 7619 | T | C | 2.723 |
| R103451 | a | 10052 | T | C | 0.444 |
| R103451 | b | 10052 | T | C | 0.394 |
| R103451 | a | 10367 | T | C | 0.160 |
| R103451 | a | 10411 | T | C | 0.403 |
| R103451 | b | 10541 | C | A | 0.244 |

Supplementary Table 5: Complete list of non-synonymous SNVs.

| ZIKV | Replicate | Position | Consensus | Variant | % | Codon pos | Consensus AA | Variant AA | Polyprotein # | Protein | AA # |
| --- | --- | --- | --- | --- | --- | --- | --- | --- | --- | --- | --- |
| MR766 | b | 221 | A | C | 0.259 | 1 | L | M | 39 | C | 39 |
| MR766 | a | 404 | A | G | 0.130 | 1 | E | K | 100 | C | 100 |
| MR766 | a | 860 | C | T | 0.268 | 1 | F | L | 252 | prM/M | 130 |
| MR766 | b | 860 | C | T | 0.388 | 1 | F | L | 252 | prM/M | 130 |
| MR766 | a | 930 | G | A | 0.274 | 2 | K | R | 275 | prM/M | 153 |
| MR766 | b | 930 | G | A | 0.237 | 2 | K | R | 275 | prM/M | 153 |
| MR766 | b | 1655 | A | G | 0.153 | 1 | A | T | 517 | E | 227 |
| MR766 | a | 1785 | T | C | 0.438 | 2 | A | V | 560 | E | 270 |
| MR766 | b | 1785 | T | C | 0.789 | 2 | A | V | 560 | E | 270 |
| MR766 | a | 1805 | G | A | 0.039 | 1 | M | V | 567 | E | 277 |
| MR766 | a | 1809 | G | A | 0.030 | 2 | D | G | 568 | E | 278 |
| MR766 | b | 2007 | G | A | 0.156 | 2 | Q | R | 634 | E | 344 |
| MR766 | b | 2232 | G | A | 0.192 | 2 | K | R | 709 | E | 419 |
| MR766 | a | 2462 | G | A | 0.379 | 1 | I | V | 786 | E | 496 |
| MR766 | b | 2462 | G | A | 0.207 | 1 | I | V | 786 | E | 496 |
| MR766 | a | 2474 | G | A | 0.071 | 1 | T | A | 790 | E | 500 |
| MR766 | a | 2490 | G | A | 0.208 | 2 | D | G | 795 | NS1 | 1 |
| MR766 | a | 2508 | G | A | 0.219 | 2 | D | G | 801 | NS1 | 7 |
| MR766 | b | 2508 | G | A | 0.047 | 2 | D | G | 801 | NS1 | 7 |
| MR766 | a | 2587 | T | G | 0.246 | 3 | K | N | 827 | NS1 | 33 |
| MR766 | b | 2644 | T | G | 0.406 | 3 | E | D | 846 | NS1 | 52 |
| MR766 | b | 2963 | T | G | 0.130 | 1 | G | W | 953 | NS1 | 159 |
| MR766 | a | 3066 | G | C | 0.291 | 2 | A | G | 987 | NS1 | 193 |
| MR766 | b | 3066 | G | C | 0.169 | 2 | A | G | 987 | NS1 | 193 |
| MR766 | b | 3095 | A | G | 0.056 | 1 | E | K | 997 | NS1 | 203 |
| MR766 | b | 3100 | A | T | 0.056 | 3 | S | R | 998 | NS1 | 204 |
| MR766 | a | 3170 | C | T | 0.396 | 1 | S | P | 1022 | NS1 | 228 |
| MR766 | b | 3170 | C | T | 0.179 | 1 | S | P | 1022 | NS1 | 228 |
| MR766 | a | 3299 | A | G | 0.361 | 1 | E | K | 1065 | NS1 | 271 |
| MR766 | b | 3299 | A | G | 0.340 | 1 | E | K | 1065 | NS1 | 271 |
| MR766 | a | 3300 | G | A | 0.769 | 2 | E | G | 1065 | NS1 | 271 |
| MR766 | b | 3300 | G | A | 0.516 | 2 | E | G | 1065 | NS1 | 271 |
| MR766 | a | 3345 | G | A | 0.522 | 2 | Y | C | 1080 | NS1 | 286 |
| MR766 | b | 3345 | G | A | 0.679 | 2 | Y | C | 1080 | NS1 | 286 |
| MR766 | a | 3402 | A | G | 0.151 | 2 | G | E | 1099 | NS1 | 305 |
| MR766 | b | 3402 | A | G | 0.114 | 2 | G | E | 1099 | NS1 | 305 |
| MR766 | a | 3408 | C | T | 0.175 | 2 | V | A | 1101 | NS1 | 307 |
| MR766 | b | 3408 | C | T | 0.099 | 2 | V | A | 1101 | NS1 | 307 |
| MR766 | b | 3417 | C | A | 0.037 | 2 | E | A | 1104 | NS1 | 310 |
| MR766 | a | 3441 | C | T | 0.175 | 2 | M | T | 1112 | NS1 | 318 |
| MR766 | b | 3441 | C | T | 0.150 | 2 | M | T | 1112 | NS1 | 318 |
| MR766 | a | 4649 | A | G | 0.059 | 1 | V | M | 1515 | NS3 | 13 |
| MR766 | a | 4650 | A | T | 0.089 | 2 | V | E | 1515 | NS3 | 13 |
| MR766 | a | 4655 | C | A | 0.061 | 1 | K | Q | 1517 | NS3 | 15 |
| MR766 | a | 4796 | A | G | 0.531 | 1 | E | K | 1564 | NS3 | 62 |
| MR766 | a | 4881 | G | A | 0.077 | 2 | D | G | 1592 | NS3 | 90 |
| MR766 | b | 4988 | A | G | 0.135 | 1 | V | I | 1628 | NS3 | 126 |
| MR766 | a | 5037 | G | A | 0.801 | 2 | K | R | 1644 | NS3 | 142 |
| MR766 | b | 5037 | G | A | 0.938 | 2 | K | R | 1644 | NS3 | 142 |
| MR766 | a | 5079 | A | T | 0.035 | 2 | I | N | 1658 | NS3 | 156 |
| MR766 | a | 5179 | C | A | 0.298 | 3 | L | F | 1691 | NS3 | 189 |
| MR766 | a | 5211 | G | A | 0.064 | 2 | K | R | 1702 | NS3 | 200 |
| MR766 | a | 5400 | G | A | 0.044 | 2 | H | R | 1765 | NS3 | 263 |
| MR766 | a | 5926 | T | G | 0.252 | 3 | E | D | 1940 | NS3 | 438 |
| MR766 | b | 5926 | T | G | 0.255 | 3 | E | D | 1940 | NS3 | 438 |
| MR766 | a | 6375 | A | G | 0.040 | 2 | R | K | 2090 | NS3 | 588 |
| MR766 | a | 6884 | A | G | 0.048 | 1 | A | T | 2260 | NS4A | 141 |
| MR766 | a | 6888 | C | T | 0.178 | 2 | V | A | 2261 | NS4A | 142 |
| MR766 | a | 6969 | A | G | 0.088 | 2 | R | K | 2288 | NS4B | 19 |
| MR766 | a | 7004 | T | G | 0.068 | 1 | D | Y | 2300 | NS4B | 31 |
| MR766 | a | 7044 | T | C | 0.068 | 2 | A | V | 2313 | NS4B | 44 |
| MR766 | b | 7046 | A | G | 0.629 | 1 | A | T | 2314 | NS4B | 45 |
| MR766 | a | 7475 | G | A | 0.031 | 1 | T | A | 2457 | NS4B | 188 |
| MR766 | a | 7478 | T | G | 0.349 | 1 | A | S | 2458 | NS4B | 189 |
| MR766 | b | 7478 | T | G | 0.881 | 1 | A | S | 2458 | NS4B | 189 |
| MR766 | b | 8049 | A | G | 0.048 | 2 | S | N | 2648 | NS5 | 128 |
| MR766 | a | 8078 | C | G | 0.057 | 1 | E | Q | 2658 | NS5 | 138 |
| MR766 | b | 8078 | C | G | 0.152 | 1 | E | Q | 2658 | NS5 | 138 |
| MR766 | a | 8175 | G | A | 0.466 | 2 | D | G | 2690 | NS5 | 170 |
| MR766 | a | 8184 | G | A | 0.172 | 2 | E | G | 2693 | NS5 | 173 |
| MR766 | b | 8398 | C | G | 0.031 | 3 | M | I | 2764 | NS5 | 244 |
| MR766 | b | 8399 | C | G | 0.041 | 1 | D | H | 2765 | NS5 | 245 |
| MR766 | a | 8586 | G | A | 0.062 | 2 | H | R | 2827 | NS5 | 307 |
| MR766 | b | 8978 | G | A | 0.021 | 1 | R | G | 2958 | NS5 | 438 |
| MR766 | a | 8990 | T | C | 0.183 | 1 | H | Y | 2962 | NS5 | 442 |
| MR766 | a | 9420 | T | C | 19.860 | 2 | A | V | 3105 | NS5 | 585 |
| MR766 | a | 9561 | T | A | 0.207 | 2 | E | V | 3152 | NS5 | 632 |
| MR766 | b | 9561 | T | A | 0.287 | 2 | E | V | 3152 | NS5 | 632 |
| MR766 | a | 9744 | T | C | 0.224 | 2 | T | I | 3213 | NS5 | 693 |
| MR766 | b | 9744 | T | C | 0.252 | 2 | T | I | 3213 | NS5 | 693 |
| MR766 | b | 10150 | G | C | 0.120 | 3 | D | E | 3348 | NS5 | 828 |
| MR766_∆E153-156_ | b | 180 | G | A | 0.512 | 2 | N | S | 25 | C | 25 |
| MR766_∆E153-156_ | a | 890 | A | G | 1.471 | 1 | V | I | 262 | prM/M | 140 |
| MR766_∆E153-156_ | b | 1347 | G | A | 0.317 | 2 | K | R | 414 | E | 124 |
| MR766_∆E153-156_ | b | 1443 | T | C | 0.218 | 2 | T | I | 446 | E | 156 |
| MR766_∆E153-156_ | b | 1913 | A | G | 0.235 | 1 | V | I | 603 | E | 313 |
| MR766_∆E153-156_ | a | 2014 | T | G | 1.154 | 3 | Q | H | 636 | E | 346 |
| MR766_∆E153-156_ | a | 2450 | G | A | 3.483 | 1 | I | V | 782 | E | 492 |
| MR766_∆E153-156_ | a | 2496 | G | A | 0.174 | 2 | D | G | 797 | NS1 | 7 |
| MR766_∆E153-156_ | b | 2858 | G | A | 1.198 | 1 | K | E | 918 | NS1 | 128 |
| MR766_∆E153-156_ | a | 3158 | C | T | 0.746 | 1 | S | P | 1018 | NS1 | 228 |
| MR766_∆E153-156_ | b | 3158 | C | T | 2.079 | 1 | S | P | 1018 | NS1 | 228 |
| MR766_∆E153-156_ | a | 3176 | A | G | 0.433 | 1 | D | N | 1024 | NS1 | 234 |
| MR766_∆E153-156_ | a | 3674 | A | G | 0.719 | 1 | G | R | 1190 | NS2A | 48 |
| MR766_∆E153-156_ | b | 4095 | G | A | 0.565 | 2 | K | R | 1330 | NS2A | 188 |
| MR766_∆E153-156_ | b | 4937 | T | C | 0.130 | 1 | P | S | 1611 | NS3 | 113 |
| MR766_∆E153-156_ | b | 4940 | T | G | 0.102 | 1 | G | * | 1612 | NS3 | 114 |
| MR766_∆E153-156_ | a | 5028 | A | G | 0.472 | 2 | C | Y | 1641 | NS3 | 143 |
| MR766_∆E153-156_ | a | 6467 | T | G | 0.122 | 1 | V | L | 2121 | NS4A | 6 |
| MR766_∆E153-156_ | a | 7157 | C | T | 0.609 | 1 | F | L | 2351 | NS4B | 86 |
| MR766_∆E153-156_ | b | 7157 | C | T | 0.556 | 1 | F | L | 2351 | NS4B | 86 |
| MR766_∆E153-156_ | a | 7497 | C | T | 0.494 | 2 | I | T | 2464 | NS4B | 199 |
| MR766_∆E153-156_ | b | 7653 | A | G | 0.108 | 2 | R | H | 2516 | NS4B | 251 |
| MR766_∆E153-156_ | b | 8574 | G | A | 0.120 | 2 | H | R | 2823 | NS5 | 307 |
| MR766_∆E153-156_ | b | 8702 | C | T | 0.437 | 1 | Y | H | 2866 | NS5 | 350 |
| MR766_∆E153-156_ | b | 9758 | G | T | 0.224 | 1 | W | G | 3218 | NS5 | 702 |
| MR766_∆E153-156_ | b | 10061 | A | G | 0.231 | 1 | G | R | 3319 | NS5 | 803 |
| MR766_∆E153-156_ | a | 10217 | T | C | 1.267 | 1 | H | Y | 3371 | NS5 | 855 |
| MR766_∆E153-156_ | b | 10217 | T | C | 1.723 | 1 | H | Y | 3371 | NS5 | 855 |
| DakAr41667 | b | 797 | C | T | 0.192 | 1 | S | P | 231 | prM/M | 109 |
| DakAr41667 | b | 1737 | T | C | 0.169 | 2 | T | I | 544 | E | 254 |
| DakAr41667 | b | 2739 | C | T | 0.224 | 2 | V | A | 878 | NS1 | 84 |
| DakAr41667 | a | 2924 | G | A | 0.219 | 1 | K | E | 940 | NS1 | 146 |
| DakAr41667 | b | 3011 | C | T | 0.812 | 1 | Y | H | 969 | NS1 | 175 |
| DakAr41667 | a | 3147 | C | T | 0.850 | 2 | M | T | 1014 | NS1 | 220 |
| DakAr41667 | b | 3147 | C | T | 0.872 | 2 | M | T | 1014 | NS1 | 220 |
| DakAr41667 | b | 3402 | A | G | 0.115 | 2 | G | E | 1099 | NS1 | 305 |
| DakAr41667 | b | 3423 | C | G | 0.152 | 2 | C | S | 1106 | NS1 | 312 |
| DakAr41667 | a | 3881 | T | C | 1.233 | 1 | L | F | 1259 | NS2A | 113 |
| DakAr41667 | a | 3905 | T | C | 0.317 | 1 | L | F | 1267 | NS2A | 121 |
| DakAr41667 | a | 4293 | T | C | 0.473 | 2 | T | I | 1396 | NS2B | 24 |
| DakAr41667 | b | 4293 | T | C | 1.547 | 2 | T | I | 1396 | NS2B | 24 |
| DakAr41667 | a | 4735 | A | G | 0.184 | 3 | M | I | 1543 | NS3 | 41 |
| DakAr41667 | b | 4735 | A | G | 0.152 | 3 | M | I | 1543 | NS3 | 41 |
| DakAr41667 | b | 6326 | C | A | 0.060 | 1 | M | L | 2074 | NS3 | 572 |
| DakAr41667 | b | 6366 | A | G | 0.062 | 2 | G | E | 2087 | NS3 | 585 |
| DakAr41667 | b | 6372 | G | A | 0.061 | 2 | K | R | 2089 | NS3 | 587 |
| DakAr41667 | b | 6375 | T | G | 0.209 | 2 | R | I | 2090 | NS3 | 588 |
| DakAr41667 | a | 7014 | C | T | 0.210 | 2 | L | P | 2303 | NS4B | 34 |
| DakAr41524 | a | 294 | C | T | 0.277 | 2 | L | P | 63 | C | 63 |
| DakAr41524 | a | 443 | A | G | 0.601 | 1 | V | I | 113 | C | 113 |
| DakAr41524 | b | 443 | A | G | 0.991 | 1 | V | I | 113 | C | 113 |
| DakAr41524 | a | 797 | C | T | 0.182 | 1 | S | P | 231 | prM/M | 109 |
| DakAr41524 | b | 797 | C | T | 0.163 | 1 | S | P | 231 | prM/M | 109 |
| DakAr41524 | a | 879 | T | C | 1.392 | 2 | A | V | 258 | prM/M | 136 |
| DakAr41524 | b | 879 | T | C | 0.807 | 2 | A | V | 258 | prM/M | 136 |
| DakAr41524 | a | 1379 | A | T | 0.215 | 1 | L | M | 425 | E | 135 |
| DakAr41524 | b | 1379 | A | T | 0.345 | 1 | L | M | 425 | E | 135 |
| DakAr41524 | a | 1433 | A | G | 0.711 | 1 | V | M | 443 | E | 153 |
| DakAr41524 | b | 1433 | A | G | 0.832 | 1 | V | M | 443 | E | 153 |
| DakAr41524 | b | 1829 | A | T | 0.206 | 1 | F | I | 575 | E | 285 |
| DakAr41524 | a | 2884 | G | T | 0.428 | 3 | S | R | 926 | NS1 | 132 |
| DakAr41524 | b | 2884 | G | T | 0.265 | 3 | S | R | 926 | NS1 | 132 |
| DakAr41524 | a | 3279 | A | T | 0.161 | 2 | V | E | 1058 | NS1 | 264 |
| DakAr41524 | b | 3279 | A | T | 0.180 | 2 | V | E | 1058 | NS1 | 264 |
| DakAr41524 | b | 3281 | G | A | 0.181 | 1 | K | E | 1059 | NS1 | 265 |
| DakAr41524 | b | 3402 | A | G | 0.211 | 2 | G | E | 1099 | NS1 | 305 |
| DakAr41524 | a | 3408 | C | T | 0.204 | 2 | V | A | 1101 | NS1 | 307 |
| DakAr41524 | a | 3423 | C | G | 0.096 | 2 | C | S | 1106 | NS1 | 312 |
| DakAr41524 | a | 3894 | T | C | 0.505 | 2 | A | V | 1263 | NS2A | 117 |
| DakAr41524 | b | 3894 | T | C | 0.887 | 2 | A | V | 1263 | NS2A | 117 |
| DakAr41524 | b | 4293 | T | C | 0.145 | 2 | A | V | 1396 | NS2B | 24 |
| DakAr41524 | a | 4362 | G | A | 0.240 | 2 | K | R | 1419 | NS2B | 47 |
| DakAr41524 | b | 4362 | G | A | 0.263 | 2 | K | R | 1419 | NS2B | 47 |
| DakAr41524 | b | 4472 | A | T | 0.045 | 1 | F | I | 1456 | NS2B | 84 |
| DakAr41524 | b | 4475 | A | T | 0.045 | 1 | S | T | 1457 | NS2B | 85 |
| DakAr41524 | b | 4593 | G | A | 0.100 | 2 | Y | C | 1496 | NS2B | 124 |
| DakAr41524 | a | 4596 | C | T | 0.044 | 2 | V | A | 1497 | NS2B | 125 |
| DakAr41524 | b | 4652 | G | A | 0.157 | 1 | K | E | 1516 | NS3 | 14 |
| DakAr41524 | b | 4658 | A | G | 0.058 | 1 | G | R | 1518 | NS3 | 16 |
| DakAr41524 | b | 5672 | T | G | 0.215 | 1 | D | Y | 1856 | NS3 | 354 |
| DakAr41524 | b | 5675 | T | C | 0.256 | 1 | H | Y | 1857 | NS3 | 355 |
| DakAr41524 | a | 6970 | C | G | 0.063 | 3 | R | S | 2288 | NS4B | 19 |
| DakAr41524 | a | 8372 | G | A | 0.253 | 1 | T | A | 2756 | NS5 | 236 |
| DakAr41524 | b | 8372 | G | A | 0.189 | 1 | T | A | 2756 | NS5 | 236 |
| DakAr41524 | a | 8471 | C | T | 6.328 | 1 | C | R | 2789 | NS5 | 269 |
| DakAr41524 | b | 8471 | C | T | 5.926 | 1 | C | R | 2789 | NS5 | 269 |
| DakAr41524 | b | 9203 | A | C | 0.059 | 1 | L | M | 3033 | NS5 | 513 |
| DakAr41524 | a | 9704 | A | C | 0.044 | 1 | L | I | 3200 | NS5 | 680 |
| FSS13025 | a | 346 | C | T | 2.042 | 2 | I | T | 80 | C | 80 |
| FSS13025 | b | 346 | C | T | 2.747 | 2 | I | T | 80 | C | 80 |
| FSS13025 | a | 609 | A | G | 1.472 | 1 | D | N | 168 | prM/M | 46 |
| FSS13025 | a | 783 | G | A | 4.569 | 1 | K | E | 226 | prM/M | 104 |
| FSS13025 | b | 783 | G | A | 0.434 | 1 | K | E | 226 | prM/M | 104 |
| FSS13025 | a | 798 | C | T | 11.550 | 1 | S | P | 231 | prM/M | 109 |
| FSS13025 | b | 798 | C | T | 6.451 | 1 | S | P | 231 | prM/M | 109 |
| FSS13025 | b | 829 | T | C | 1.023 | 2 | T | I | 241 | prM/M | 119 |
| FSS13025 | a | 861 | C | T | 0.380 | 1 | F | L | 252 | prM/M | 130 |
| FSS13025 | b | 861 | C | T | 0.645 | 1 | F | L | 252 | prM/M | 130 |
| FSS13025 | a | 906 | T | C | 2.385 | 1 | L | F | 267 | prM/M | 145 |
| FSS13025 | b | 906 | T | C | 0.754 | 1 | L | F | 267 | prM/M | 145 |
| FSS13025 | a | 937 | C | T | 0.947 | 2 | I | T | 277 | prM/M | 155 |
| FSS13025 | b | 1126 | C | T | 0.494 | 2 | V | A | 340 | E | 50 |
| FSS13025 | a | 1434 | A | G | 0.600 | 1 | V | I | 443 | E | 153 |
| FSS13025 | b | 1921 | T | C | 1.022 | 2 | T | I | 605 | E | 315 |
| FSS13025 | b | 2479 | T | C | 0.256 | 2 | A | V | 791 | E | 501 |
| FSS13025 | b | 2775 | A | C | 0.026 | 1 | P | T | 890 | NS1 | 96 |
| FSS13025 | b | 2776 | T | C | 0.071 | 2 | P | L | 890 | NS1 | 96 |
| FSS13025 | b | 2797 | A | G | 0.973 | 2 | R | K | 897 | NS1 | 103 |
| FSS13025 | b | 2925 | G | A | 2.649 | 1 | K | E | 940 | NS1 | 146 |
| FSS13025 | a | 3147 | G | A | 1.598 | 1 | M | V | 1014 | NS1 | 220 |
| FSS13025 | b | 3147 | G | A | 1.877 | 1 | M | V | 1014 | NS1 | 220 |
| FSS13025 | a | 3149 | T | G | 0.247 | 3 | M | I | 1014 | NS1 | 220 |
| FSS13025 | b | 3149 | A | G | 1.396 | 3 | M | I | 1014 | NS1 | 220 |
| FSS13025 | b | 3149 | T | G | 0.188 | 3 | M | I | 1014 | NS1 | 220 |
| FSS13025 | a | 3170 | T | G | 0.412 | 3 | K | N | 1021 | NS1 | 227 |
| FSS13025 | b | 3170 | T | G | 0.162 | 3 | K | N | 1021 | NS1 | 227 |
| FSS13025 | a | 3193 | A | G | 0.614 | 2 | G | E | 1029 | NS1 | 235 |
| FSS13025 | a | 3223 | G | A | 0.244 | 2 | K | R | 1039 | NS1 | 245 |
| FSS13025 | a | 4338 | G | A | 0.526 | 1 | I | V | 1411 | NS2B | 39 |
| FSS13025 | b | 4338 | G | A | 0.459 | 1 | I | V | 1411 | NS2B | 39 |
| FSS13025 | b | 4536 | A | G | 35.940 | 1 | A | T | 1477 | NS2B | 105 |
| FSS13025 | b | 5872 | T | C | 0.130 | 2 | A | V | 1922 | NS3 | 420 |
| FSS13025 | b | 5875 | G | A | 0.102 | 2 | D | G | 1923 | NS3 | 421 |
| FSS13025 | b | 5982 | T | A | 0.116 | 1 | R | W | 1959 | NS3 | 457 |
| FSS13025 | b | 6363 | C | T | 0.237 | 1 | Y | H | 2086 | NS3 | 584 |
| FSS13025 | a | 6373 | G | A | 0.656 | 2 | K | R | 2089 | NS3 | 587 |
| FSS13025 | b | 6373 | G | A | 1.059 | 2 | K | R | 2089 | NS3 | 587 |
| FSS13025 | b | 6696 | G | A | 1.464 | 1 | I | V | 2197 | NS4A | 78 |
| FSS13025 | b | 6984 | A | G | 0.307 | 1 | A | T | 2293 | NS4B | 24 |
| FSS13025 | a | 7005 | T | G | 0.073 | 1 | D | Y | 2300 | NS4B | 31 |
| FSS13025 | a | 7602 | G | A | 3.579 | 1 | S | G | 2499 | NS4B | 230 |
| FSS13025 | b | 7602 | G | A | 3.682 | 1 | S | G | 2499 | NS4B | 230 |
| FSS13025 | b | 7813 | T | C | 2.908 | 2 | A | V | 2569 | NS5 | 49 |
| FSS13025 | b | 8185 | G | A | 0.216 | 2 | E | G | 2693 | NS5 | 173 |
| FSS13025 | a | 8568 | C | T | 0.067 | 1 | Y | H | 2821 | NS5 | 301 |
| FSS13025 | b | 8568 | C | T | 0.043 | 1 | Y | H | 2821 | NS5 | 301 |
| FSS13025 | a | 9003 | A | G | 0.096 | 1 | G | R | 2966 | NS5 | 446 |
| FSS13025 | b | 9159 | A | T | 0.035 | 1 | W | R | 3018 | NS5 | 498 |
| FSS13025 | b | 9160 | T | G | 0.026 | 2 | W | L | 3018 | NS5 | 498 |
| FSS13025 | b | 10174 | T | C | 0.043 | 2 | T | I | 3356 | NS5 | 836 |
| FSS13025 | a | 10230 | T | C | 3.301 | 1 | H | Y | 3375 | NS5 | 855 |
| FSS13025 | b | 10230 | T | C | 3.191 | 1 | H | Y | 3375 | NS5 | 855 |
| PA259249 | a | 448 | A | G | 0.224 | 2 | G | D | 114 | C | 114 |
| PA259249 | a | 463 | T | C | 0.292 | 2 | T | I | 119 | C | 119 |
| PA259249 | a | 468 | G | A | 0.243 | 1 | M | V | 121 | C | 121 |
| PA259249 | a | 679 | C | G | 0.086 | 2 | C | S | 191 | prM/M | 69 |
| PA259249 | a | 793 | T | C | 9.420 | 2 | T | M | 229 | prM/M | 107 |
| PA259249 | b | 793 | T | C | 6.695 | 2 | T | M | 229 | prM/M | 107 |
| PA259249 | a | 816 | C | T | 0.805 | 1 | S | P | 237 | prM/M | 115 |
| PA259249 | b | 816 | C | T | 0.722 | 1 | S | P | 237 | prM/M | 115 |
| PA259249 | a | 829 | T | C | 0.894 | 2 | T | I | 241 | prM/M | 119 |
| PA259249 | b | 829 | T | C | 2.235 | 2 | T | I | 241 | prM/M | 119 |
| PA259249 | b | 834 | T | C | 0.440 | 1 | H | Y | 243 | prM/M | 121 |
| PA259249 | a | 876 | C | T | 0.797 | 1 | F | L | 257 | prM/M | 135 |
| PA259249 | b | 876 | C | T | 0.249 | 1 | F | L | 257 | prM/M | 135 |
| PA259249 | a | 878 | A | C | 0.344 | 3 | F | L | 257 | prM/M | 135 |
| PA259249 | a | 927 | A | C | 0.157 | 1 | Q | K | 274 | prM/M | 152 |
| PA259249 | a | 973 | G | A | 0.302 | 2 | Y | C | 289 | prM/M | 167 |
| PA259249 | a | 1120 | T | C | 0.164 | 2 | T | I | 338 | E | 48 |
| PA259249 | b | 1126 | C | T | 0.316 | 2 | V | A | 340 | E | 50 |
| PA259249 | a | 1218 | C | T | 0.309 | 1 | Y | H | 371 | E | 81 |
| PA259249 | b | 1218 | C | T | 0.368 | 1 | Y | H | 371 | E | 81 |
| PA259249 | b | 1240 | T | C | 0.476 | 2 | T | I | 378 | E | 88 |
| PA259249 | a | 1270 | G | A | 0.045 | 2 | D | G | 388 | E | 98 |
| PA259249 | a | 1330 | G | A | 0.356 | 2 | K | R | 408 | E | 118 |
| PA259249 | b | 1656 | C | G | 0.898 | 1 | A | P | 517 | E | 227 |
| PA259249 | a | 1786 | T | C | 0.224 | 2 | A | V | 560 | E | 270 |
| PA259249 | a | 2149 | C | T | 0.396 | 2 | V | A | 681 | E | 391 |
| PA259249 | a | 2178 | T | C | 0.395 | 1 | H | Y | 691 | E | 401 |
| PA259249 | b | 2178 | T | C | 0.446 | 1 | H | Y | 691 | E | 401 |
| PA259249 | a | 2242 | T | C | 0.224 | 2 | A | V | 712 | E | 422 |
| PA259249 | a | 2305 | G | A | 0.224 | 2 | K | R | 733 | E | 443 |
| PA259249 | a | 2341 | T | C | 0.381 | 2 | S | L | 745 | E | 455 |
| PA259249 | a | 2389 | C | T | 0.214 | 2 | L | S | 761 | E | 471 |
| PA259249 | b | 2389 | C | T | 0.279 | 2 | L | S | 761 | E | 471 |
| PA259249 | a | 2478 | A | G | 0.250 | 1 | A | T | 791 | E | 501 |
| PA259249 | b | 2478 | A | G | 0.229 | 1 | A | T | 791 | E | 501 |
| PA259249 | a | 2662 | C | T | 7.720 | 2 | F | S | 852 | NS1 | 58 |
| PA259249 | b | 2767 | C | T | 0.399 | 2 | V | A | 887 | NS1 | 93 |
| PA259249 | a | 2781 | C | T | 0.611 | 1 | W | R | 892 | NS1 | 98 |
| PA259249 | b | 2781 | C | T | 0.377 | 1 | W | R | 892 | NS1 | 98 |
| PA259249 | a | 2782 | C | G | 0.153 | 2 | W | S | 892 | NS1 | 98 |
| PA259249 | a | 2783 | T | G | 0.224 | 3 | W | C | 892 | NS1 | 98 |
| PA259249 | b | 2783 | T | G | 0.243 | 3 | W | C | 892 | NS1 | 98 |
| PA259249 | a | 2797 | A | G | 3.050 | 2 | R | K | 897 | NS1 | 103 |
| PA259249 | b | 2797 | A | G | 2.587 | 2 | R | K | 897 | NS1 | 103 |
| PA259249 | a | 2871 | G | A | 10.400 | 1 | K | E | 922 | NS1 | 128 |
| PA259249 | b | 2871 | G | A | 6.611 | 1 | K | E | 922 | NS1 | 128 |
| PA259249 | a | 2899 | A | G | 0.586 | 2 | G | D | 931 | NS1 | 137 |
| PA259249 | b | 2899 | A | G | 0.351 | 2 | G | D | 931 | NS1 | 137 |
| PA259249 | a | 2905 | T | C | 0.661 | 2 | T | I | 933 | NS1 | 139 |
| PA259249 | b | 2905 | T | C | 0.440 | 2 | T | I | 933 | NS1 | 139 |
| PA259249 | a | 2910 | G | A | 1.032 | 1 | K | E | 935 | NS1 | 141 |
| PA259249 | b | 2910 | G | A | 0.753 | 1 | K | E | 935 | NS1 | 141 |
| PA259249 | a | 2914 | G | A | 0.408 | 2 | E | G | 936 | NS1 | 142 |
| PA259249 | b | 2914 | G | A | 0.277 | 2 | E | G | 936 | NS1 | 142 |
| PA259249 | a | 2923 | C | T | 0.799 | 2 | L | P | 939 | NS1 | 145 |
| PA259249 | b | 2923 | C | T | 1.069 | 2 | L | P | 939 | NS1 | 145 |
| PA259249 | a | 3012 | C | T | 0.330 | 1 | Y | H | 969 | NS1 | 175 |
| PA259249 | a | 3019 | C | T | 0.492 | 2 | L | S | 971 | NS1 | 177 |
| PA259249 | b | 3019 | C | T | 0.607 | 2 | L | S | 971 | NS1 | 177 |
| PA259249 | a | 3110 | A | T | 0.523 | 3 | N | K | 1001 | NS1 | 207 |
| PA259249 | b | 3110 | A | T | 0.420 | 3 | N | K | 1001 | NS1 | 207 |
| PA259249 | a | 3147 | G | A | 0.903 | 1 | M | V | 1014 | NS1 | 220 |
| PA259249 | b | 3147 | G | A | 0.427 | 1 | M | V | 1014 | NS1 | 220 |
| PA259249 | a | 3148 | C | T | 3.662 | 2 | M | T | 1014 | NS1 | 220 |
| PA259249 | b | 3148 | C | T | 2.254 | 2 | M | T | 1014 | NS1 | 220 |
| PA259249 | a | 3149 | A | G | 10.230 | 3 | M | I | 1014 | NS1 | 220 |
| PA259249 | b | 3149 | A | G | 1.489 | 3 | M | I | 1014 | NS1 | 220 |
| PA259249 | a | 3230 | C | A | 0.540 | 3 | L | F | 1041 | NS1 | 247 |
| PA259249 | b | 3230 | C | A | 0.459 | 3 | L | F | 1041 | NS1 | 247 |
| PA259249 | a | 3282 | G | A | 4.128 | 1 | K | E | 1059 | NS1 | 265 |
| PA259249 | b | 3282 | G | A | 3.168 | 1 | K | E | 1059 | NS1 | 265 |
| PA259249 | a | 3399 | G | A | 0.293 | 1 | S | G | 1098 | NS1 | 304 |
| PA259249 | a | 3400 | A | G | 1.302 | 2 | S | N | 1098 | NS1 | 304 |
| PA259249 | b | 3400 | A | G | 0.565 | 2 | S | N | 1098 | NS1 | 304 |
| PA259249 | a | 3406 | A | G | 0.537 | 2 | R | K | 1100 | NS1 | 306 |
| PA259249 | b | 3406 | A | G | 0.343 | 2 | R | K | 1100 | NS1 | 306 |
| PA259249 | a | 3411 | G | A | 0.278 | 1 | I | V | 1102 | NS1 | 308 |
| PA259249 | b | 3465 | G | A | 0.398 | 1 | K | E | 1120 | NS1 | 326 |
| PA259249 | b | 3510 | T | C | 0.307 | 1 | P | S | 1135 | NS1 | 341 |
| PA259249 | a | 3513 | A | G | 0.426 | 1 | E | K | 1136 | NS1 | 342 |
| PA259249 | b | 3513 | A | G | 0.314 | 1 | E | K | 1136 | NS1 | 342 |
| PA259249 | a | 3514 | G | A | 1.880 | 2 | E | G | 1136 | NS1 | 342 |
| PA259249 | b | 3514 | G | A | 1.543 | 2 | E | G | 1136 | NS1 | 342 |
| PA259249 | a | 3535 | A | T | 0.283 | 2 | M | K | 1143 | NS1 | 349 |
| PA259249 | a | 3828 | C | T | 0.291 | 1 | F | L | 1241 | NS2A | 95 |
| PA259249 | a | 3894 | A | G | 1.101 | 1 | A | T | 1263 | NS2A | 117 |
| PA259249 | b | 3894 | A | G | 0.778 | 1 | A | T | 1263 | NS2A | 117 |
| PA259249 | a | 3895 | T | C | 1.193 | 2 | A | V | 1263 | NS2A | 117 |
| PA259249 | b | 3895 | T | C | 0.919 | 2 | A | V | 1263 | NS2A | 117 |
| PA259249 | a | 3898 | C | T | 0.656 | 2 | I | T | 1264 | NS2A | 118 |
| PA259249 | b | 4015 | T | C | 1.728 | 2 | A | V | 1303 | NS2A | 157 |
| PA259249 | b | 4294 | T | C | 0.251 | 2 | A | V | 1396 | NS2B | 24 |
| PA259249 | a | 4296 | A | G | 0.690 | 1 | D | N | 1397 | NS2B | 25 |
| PA259249 | b | 4296 | A | G | 0.517 | 1 | D | N | 1397 | NS2B | 25 |
| PA259249 | a | 4319 | A | G | 1.485 | 3 | M | I | 1404 | NS2B | 32 |
| PA259249 | b | 4319 | A | G | 1.376 | 3 | M | I | 1404 | NS2B | 32 |
| PA259249 | a | 4341 | A | G | 0.409 | 1 | V | I | 1412 | NS2B | 40 |
| PA259249 | a | 4713 | G | A | 0.422 | 1 | T | A | 1536 | NS3 | 34 |
| PA259249 | b | 4713 | G | A | 0.264 | 1 | T | A | 1536 | NS3 | 34 |
| PA259249 | a | 4827 | A | G | 0.060 | 1 | V | I | 1574 | NS3 | 72 |
| PA259249 | a | 4914 | G | C | 0.254 | 1 | P | A | 1603 | NS3 | 101 |
| PA259249 | a | 4933 | A | G | 0.211 | 2 | R | K | 1609 | NS3 | 107 |
| PA259249 | b | 4933 | A | G | 0.258 | 2 | R | K | 1609 | NS3 | 107 |
| PA259249 | a | 5041 | A | G | 0.279 | 2 | C | Y | 1645 | NS3 | 143 |
| PA259249 | b | 5041 | A | G | 0.489 | 2 | C | Y | 1645 | NS3 | 143 |
| PA259249 | a | 5395 | G | T | 0.065 | 2 | M | R | 1763 | NS3 | 261 |
| PA259249 | a | 5448 | C | T | 0.112 | 1 | Y | H | 1781 | NS3 | 279 |
| PA259249 | a | 5676 | T | C | 1.692 | 1 | H | Y | 1857 | NS3 | 355 |
| PA259249 | b | 5676 | T | C | 1.072 | 1 | H | Y | 1857 | NS3 | 355 |
| PA259249 | a | 5680 | T | C | 1.550 | 2 | S | F | 1858 | NS3 | 356 |
| PA259249 | b | 5680 | T | C | 1.231 | 2 | S | F | 1858 | NS3 | 356 |
| PA259249 | a | 5793 | A | G | 0.183 | 1 | E | K | 1896 | NS3 | 394 |
| PA259249 | a | 6894 | T | C | 0.241 | 1 | L | F | 2263 | NS4A | 144 |
| PA259249 | a | 6955 | T | G | 0.188 | 2 | S | I | 2283 | NS4B | 14 |
| PA259249 | b | 6955 | T | G | 0.337 | 2 | S | I | 2283 | NS4B | 14 |
| PA259249 | b | 7968 | G | A | 0.134 | 1 | K | E | 2621 | NS5 | 101 |
| PA259249 | a | 8472 | C | T | 0.596 | 1 | C | R | 2789 | NS5 | 269 |
| PA259249 | b | 8472 | C | T | 0.610 | 1 | C | R | 2789 | NS5 | 269 |
| PA259249 | b | 8568 | C | T | 0.067 | 1 | Y | H | 2821 | NS5 | 301 |
| PA259249 | b | 9010 | C | G | 0.128 | 2 | C | S | 2968 | NS5 | 448 |
| PA259249 | a | 9406 | G | A | 0.360 | 2 | K | R | 3100 | NS5 | 580 |
| PA259249 | b | 9406 | G | A | 0.288 | 2 | K | R | 3100 | NS5 | 580 |
| PA259249 | a | 9966 | T | C | 0.278 | 1 | L | F | 3287 | NS5 | 767 |
| PA259249 | b | 9966 | T | C | 0.341 | 1 | L | F | 3287 | NS5 | 767 |
| PA259249 | a | 10192 | A | G | 0.200 | 2 | G | E | 3362 | NS5 | 842 |
| R103451 | a | 436 | C | T | 0.326 | 2 | V | A | 110 | C | 110 |
| R103451 | b | 436 | C | T | 0.495 | 2 | V | A | 110 | C | 110 |
| R103451 | a | 612 | A | G | 0.432 | 1 | A | T | 169 | prM/M | 47 |
| R103451 | b | 612 | A | G | 0.359 | 1 | A | T | 169 | prM/M | 47 |
| R103451 | a | 721 | G | A | 0.262 | 2 | H | R | 205 | prM/M | 83 |
| R103451 | b | 721 | G | A | 0.407 | 2 | H | R | 205 | prM/M | 83 |
| R103451 | b | 748 | A | G | 0.168 | 2 | R | K | 214 | prM/M | 92 |
| R103451 | a | 834 | T | C | 0.196 | 1 | H | Y | 243 | prM/M | 121 |
| R103451 | a | 861 | C | T | 0.415 | 1 | F | L | 252 | prM/M | 130 |
| R103451 | b | 861 | C | T | 0.374 | 1 | F | L | 252 | prM/M | 130 |
| R103451 | b | 950 | A | G | 0.125 | 3 | M | I | 281 | prM/M | 159 |
| R103451 | a | 960 | T | A | 1.643 | 1 | I | F | 285 | prM/M | 163 |
| R103451 | b | 960 | T | A | 1.316 | 1 | I | F | 285 | prM/M | 163 |
| R103451 | a | 1114 | C | T | 0.448 | 2 | V | A | 336 | E | 46 |
| R103451 | a | 1120 | T | C | 0.163 | 2 | T | I | 338 | E | 48 |
| R103451 | b | 1921 | T | C | 0.427 | 2 | T | I | 605 | E | 315 |
| R103451 | a | 1933 | T | C | 0.199 | 2 | A | V | 609 | E | 319 |
| R103451 | a | 2178 | T | C | 0.412 | 1 | H | Y | 691 | E | 401 |
| R103451 | b | 2178 | T | C | 0.340 | 1 | H | Y | 691 | E | 401 |
| R103451 | a | 2242 | T | C | 0.173 | 2 | A | V | 712 | E | 422 |
| R103451 | a | 2305 | G | A | 25.750 | 2 | K | R | 733 | E | 443 |
| R103451 | a | 2341 | T | C | 0.193 | 2 | S | L | 745 | E | 455 |
| R103451 | b | 2462 | T | G | 0.277 | 3 | L | F | 785 | E | 495 |
| R103451 | a | 2478 | A | G | 0.240 | 1 | A | T | 791 | E | 501 |
| R103451 | b | 2478 | A | G | 0.302 | 1 | A | T | 791 | E | 501 |
| R103451 | a | 2769 | C | A | 1.219 | 1 | K | Q | 888 | NS1 | 94 |
| R103451 | b | 2769 | C | A | 1.041 | 1 | K | Q | 888 | NS1 | 94 |
| R103451 | b | 2949 | G | C | 0.048 | 1 | L | V | 948 | NS1 | 154 |
| R103451 | a | 3013 | A | C | 3.187 | 2 | S | Y | 969 | NS1 | 175 |
| R103451 | b | 3013 | A | C | 2.704 | 2 | S | Y | 969 | NS1 | 175 |
| R103451 | b | 3620 | T | G | 0.080 | 3 | K | N | 1171 | NS2A | 25 |
| R103451 | a | 3669 | A | G | 0.395 | 1 | V | I | 1188 | NS2A | 42 |
| R103451 | b | 3669 | A | G | 0.433 | 1 | V | I | 1188 | NS2A | 42 |
| R103451 | a | 4215 | G | A | 3.188 | 1 | R | G | 1370 | NS2A | 224 |
| R103451 | b | 4215 | G | A | 2.624 | 1 | R | G | 1370 | NS2A | 224 |
| R103451 | a | 4326 | A | G | 0.483 | 1 | V | I | 1407 | NS2B | 35 |
| R103451 | b | 4326 | A | G | 0.900 | 1 | V | I | 1407 | NS2B | 35 |
| R103451 | b | 5797 | C | T | 0.189 | 2 | F | S | 1897 | NS3 | 395 |
| R103451 | a | 5872 | T | C | 0.040 | 2 | A | V | 1922 | NS3 | 420 |
| R103451 | a | 5875 | G | A | 0.032 | 2 | D | G | 1923 | NS3 | 421 |
| R103451 | a | 6361 | A | G | 0.250 | 2 | R | K | 2085 | NS3 | 583 |
| R103451 | b | 6361 | A | G | 0.362 | 2 | R | K | 2085 | NS3 | 583 |
| R103451 | a | 6971 | T | G | 0.071 | 3 | R | S | 2288 | NS4B | 19 |
| R103451 | a | 6973 | A | G | 0.175 | 2 | R | K | 2289 | NS4B | 20 |
| R103451 | a | 7173 | C | T | 0.292 | 1 | Y | H | 2356 | NS4B | 87 |
| R103451 | a | 7894 | C | A | 0.405 | 2 | K | T | 2596 | NS5 | 76 |
| R103451 | b | 7894 | C | A | 0.313 | 2 | K | T | 2596 | NS5 | 76 |
| R103451 | a | 9010 | C | G | 0.144 | 2 | C | S | 2968 | NS5 | 448 |
| R103451 | b | 9010 | C | G | 0.130 | 2 | C | S | 2968 | NS5 | 448 |
| R103451 | a | 9424 | G | A | 0.200 | 2 | E | G | 3106 | NS5 | 586 |
| R103451 | b | 9424 | G | A | 0.207 | 2 | E | G | 3106 | NS5 | 586 |
| R103451 | a | 9724 | C | T | 0.156 | 2 | M | T | 3206 | NS5 | 686 |
| R103451 | b | 9727 | A | G | 0.060 | 2 | G | E | 3207 | NS5 | 687 |
| R103451 | a | 10017 | G | T | 0.382 | 1 | S | A | 3304 | NS5 | 784 |
| R103451 | b | 10017 | G | T | 0.391 | 1 | S | A | 3304 | NS5 | 784 |

Supplementary Table 6. SNVs shared among strains sequenced in this study and data downloaded from SRA database.

| ZIKV | Replicate* | Position | Consensus | Variant | % | Syn / Non-syn^ | Codon position | Consensus AA | Variant AA | Polyprotein # | Protein | AA # |
| --- | --- | --- | --- | --- | --- | --- | --- | --- | --- | --- | --- | --- |
| PA259249 | #1 | 490 | G | A | 0.59 | Non-syn | 2 | R | H | 128 | prM/M | 6 |
| PA259249 | #1 | 491 | C | T | 6.19 | Syn | 3 | R | R | 128 | prM/M | 6 |
| PA259249 | a | 491 | C | T | 7.47 | Syn | 3 | R | R | 128 | prM/M | 6 |
| PA259249 | b | 491 | C | T | 3.19 | Syn | 3 | R | R | 128 | prM/M | 6 |
| PA259249 | a | 816 | T | C | 0.81 | Non-syn | 1 | S | P | 237 | prM/M | 115 |
| PA259249 | b | 816 | T | C | 0.72 | Non-syn | 1 | S | P | 237 | prM/M | 115 |
| PA259249 | #1 | 817 | C | G | 4.00 | Non-syn | 2 | S | * | 237 | prM/M | 115 |
| MR766 | a | 1099 | T | C | 2.20 | Syn | 3 | V | V | 331 | E | 41 |
| MR766 | b | 1099 | T | C | 2.61 | Syn | 3 | V | V | 331 | E | 41 |
| MR766_∆E153-156_ | #4 | 1099 | T | C | 42.25 | Syn | 3 | V | V | 331 | E | 41 |
| FSS13025 | b | 1921 | C | T | 1.02 | Non-syn | 2 | T | I | 605 | E | 315 |
| R103451 | #5 | 1921 | C | T | 3.23 | Non-syn | 2 | T | I | 605 | E | 315 |
| R103451 | b | 1921 | C | T | 0.43 | Non-syn | 2 | T | I | 605 | E | 315 |
| PA259249 | a | 2305 | A | G | 0.22 | Non-syn | 2 | K | R | 733 | E | 443 |
| R103451 | #5 | 2305 | G | A | 25.06 | Non-syn | 2 | R | K | 733 | E | 443 |
| R103451 | a | 2305 | A | G | 25.75 | Non-syn | 2 | K | R | 733 | E | 443 |
| MR766 | a | 2587 | G | T | 0.25 | Non-syn | 3 | K | N | 827 | NS1 | 33 |
| PA259249 | #1 | 2587 | A | G | 0.35 | Non-syn | 2 | K | R | 827 | NS1 | 33 |
| PA259249 | #1 | 2588 | G | A | 0.35 | Syn | 3 | K | K | 827 | NS1 | 33 |
| PA259249 | #1 | 2662 | T | C | 3.89 | Non-syn | 2 | F | S | 852 | NS1 | 58 |
| PA259249 | a | 2662 | T | C | 7.72 | Non-syn | 2 | F | S | 852 | NS1 | 58 |
| PA259249 | a | 2663 | C | T | 0.25 | Syn | 3 | F | F | 852 | NS1 | 58 |
| PA259249 | #1 | 2871 | A | G | 3.17 | Non-syn | 1 | K | E | 922 | NS1 | 128 |
| PA259249 | a | 2871 | A | G | 10.40 | Non-syn | 1 | K | E | 922 | NS1 | 128 |
| PA259249 | b | 2871 | A | G | 6.61 | Non-syn | 1 | K | E | 922 | NS1 | 128 |
| MR766 | a | 2923 | T | C | 0.30 | Syn | 3 | L | L | 939 | NS1 | 145 |
| MR766 | b | 2923 | T | C | 0.29 | Syn | 3 | L | L | 939 | NS1 | 145 |
| PA259249 | #1 | 2923 | T | C | 1.88 | Non-syn | 2 | L | P | 939 | NS1 | 145 |
| PA259249 | a | 2923 | T | C | 0.80 | Non-syn | 2 | L | P | 939 | NS1 | 145 |
| PA259249 | b | 2923 | T | C | 1.07 | Non-syn | 2 | L | P | 939 | NS1 | 145 |
| PA259249 | #1 | 3019 | T | C | 1.95 | Non-syn | 2 | L | S | 971 | NS1 | 177 |
| PA259249 | a | 3019 | T | C | 0.49 | Non-syn | 2 | L | S | 971 | NS1 | 177 |
| PA259249 | b | 3019 | T | C | 0.61 | Non-syn | 2 | L | S | 971 | NS1 | 177 |
| DakAr41667 | a | 3147 | T | C | 0.85 | Non-syn | 2 | M | T | 1014 | NS1 | 220 |
| DakAr41667 | b | 3147 | T | C | 0.87 | Non-syn | 2 | M | T | 1014 | NS1 | 220 |
| FSS13025 | a | 3147 | A | G | 1.60 | Non-syn | 1 | M | V | 1014 | NS1 | 220 |
| FSS13025 | b | 3147 | A | G | 1.88 | Non-syn | 1 | M | V | 1014 | NS1 | 220 |
| PA259249 | a | 3147 | A | G | 0.90 | Non-syn | 1 | M | V | 1014 | NS1 | 220 |
| PA259249 | b | 3147 | A | G | 0.43 | Non-syn | 1 | M | V | 1014 | NS1 | 220 |
| PA259249 | #1 | 3148 | T | C | 6.12 | Non-syn | 2 | M | T | 1014 | NS1 | 220 |
| PA259249 | a | 3148 | T | C | 3.66 | Non-syn | 2 | M | T | 1014 | NS1 | 220 |
| PA259249 | b | 3148 | T | C | 2.25 | Non-syn | 2 | M | T | 1014 | NS1 | 220 |
| FSS13025 | a | 3149 | G | T | 0.25 | Non-syn | 3 | M | I | 1014 | NS1 | 220 |
| FSS13025 | b | 3149 | G | T | 0.19 | Non-syn | 3 | M | I | 1014 | NS1 | 220 |
| FSS13025 | b | 3149 | G | A | 1.40 | Non-syn | 3 | M | I | 1014 | NS1 | 220 |
| PA259249 | a | 3149 | G | A | 10.23 | Non-syn | 3 | M | I | 1014 | NS1 | 220 |
| PA259249 | b | 3149 | G | A | 1.49 | Non-syn | 3 | M | I | 1014 | NS1 | 220 |
| PA259249 | #1 | 3513 | G | T | 1.70 | Non-syn | 1 | E | * | 1136 | NS1 | 342 |
| PA259249 | a | 3513 | G | A | 0.43 | Non-syn | 1 | E | K | 1136 | NS1 | 342 |
| PA259249 | b | 3513 | G | A | 0.31 | Non-syn | 1 | E | K | 1136 | NS1 | 342 |
| R103451 | #5 | 3513 | G | T | 2.31 | Non-syn | 1 | E | * | 1136 | NS1 | 342 |
| PA259249 | a | 3514 | A | G | 1.88 | Non-syn | 2 | E | G | 1136 | NS1 | 342 |
| PA259249 | b | 3514 | A | G | 1.54 | Non-syn | 2 | E | G | 1136 | NS1 | 342 |
| PA259249 | #1 | 3515 | A | G | 0.26 | Syn | 3 | E | E | 1136 | NS1 | 342 |
| PA259249 | #1 | 3619 | A | G | 0.88 | Non-syn | 2 | K | R | 1171 | NS2A | 25 |
| PA259249 | #1 | 3619 | A | T | 1.24 | Non-syn | 2 | K | M | 1171 | NS2A | 25 |
| R103451 | #5 | 3619 | A | T | 4.53 | Non-syn | 2 | K | M | 1171 | NS2A | 25 |
| R103451 | b | 3620 | G | T | 0.08 | Non-syn | 3 | K | N | 1171 | NS2A | 25 |
| DakAr41524 | #6 | 3894 | C | T | 7.03 | Non-syn | 2 | A | V | 1263 | NS2A | 117 |
| DakAr41524 | a | 3894 | C | T | 0.51 | Non-syn | 2 | A | V | 1263 | NS2A | 117 |
| DakAr41524 | b | 3894 | C | T | 0.89 | Non-syn | 2 | A | V | 1263 | NS2A | 117 |
| PA259249 | #1 | 3894 | G | A | 1.28 | Non-syn | 1 | A | T | 1263 | NS2A | 117 |
| PA259249 | a | 3894 | G | A | 1.10 | Non-syn | 1 | A | T | 1263 | NS2A | 117 |
| PA259249 | b | 3894 | G | A | 0.78 | Non-syn | 1 | A | T | 1263 | NS2A | 117 |
| PA259249 | #1 | 3895 | C | T | 20.32 | Non-syn | 2 | A | V | 1263 | NS2A | 117 |
| PA259249 | a | 3895 | C | T | 1.19 | Non-syn | 2 | A | V | 1263 | NS2A | 117 |
| PA259249 | b | 3895 | C | T | 0.92 | Non-syn | 2 | A | V | 1263 | NS2A | 117 |
| DakAr41524 | #2 | 3905 | C | T | 4.48 | Non-syn | 1 | L | F | 1267 | NS2A | 121 |
| DakAr41667 | a | 3905 | C | T | 0.32 | Non-syn | 1 | L | F | 1267 | NS2A | 121 |
| PA259249 | #1 | 4015 | C | T | 2.05 | Non-syn | 2 | A | V | 1303 | NS2A | 157 |
| PA259249 | b | 4015 | C | T | 1.73 | Non-syn | 2 | A | V | 1303 | NS2A | 157 |
| R103451 | #5 | 4215 | A | G | 1.67 | Non-syn | 1 | R | G | 1370 | NS2A | 224 |
| R103451 | a | 4215 | A | G | 3.19 | Non-syn | 1 | R | G | 1370 | NS2A | 224 |
| R103451 | b | 4215 | A | G | 2.62 | Non-syn | 1 | R | G | 1370 | NS2A | 224 |
| PA259249 | #1 | 4317 | A | G | 0.25 | Non-syn | 1 | M | V | 1404 | NS2B | 32 |
| PA259249 | a | 4319 | G | A | 1.49 | Non-syn | 3 | M | I | 1404 | NS2B | 32 |
| PA259249 | b | 4319 | G | A | 1.38 | Non-syn | 3 | M | I | 1404 | NS2B | 32 |
| DakAr41524 | #2 | 4535 | G | A | 0.31 | Non-syn | 1 | A | T | 1477 | NS2B | 105 |
| FSS13025 | b | 4536 | G | A | 35.94 | Non-syn | 1 | A | T | 1477 | NS2B | 105 |
| DakAr41524 | a | 4537 | C | T | 0.21 | Syn | 3 | A | A | 1477 | NS2B | 105 |
| DakAr41524 | b | 4537 | C | T | 0.18 | Syn | 3 | A | A | 1477 | NS2B | 105 |
| PA259249 | a | 4914 | C | G | 0.25 | Non-syn | 1 | P | A | 1603 | NS3 | 101 |
| PA259249 | #1 | 4915 | C | A | 0.40 | Non-syn | 2 | P | H | 1603 | NS3 | 101 |
| PA259249 | #1 | 4933 | G | A | 1.23 | Non-syn | 2 | R | K | 1609 | NS3 | 107 |
| PA259249 | a | 4933 | G | A | 0.21 | Non-syn | 2 | R | K | 1609 | NS3 | 107 |
| PA259249 | b | 4933 | G | A | 0.26 | Non-syn | 2 | R | K | 1609 | NS3 | 107 |
| DakAr41524 | b | 5675 | C | T | 0.26 | Non-syn | 1 | H | Y | 1857 | NS3 | 355 |
| PA259249 | #1 | 5676 | C | T | 3.05 | Non-syn | 1 | H | Y | 1857 | NS3 | 355 |
| PA259249 | a | 5676 | C | T | 1.07 | Non-syn | 1 | H | Y | 1857 | NS3 | 355 |
| PA259249 | b | 5676 | C | T | 1.69 | Non-syn | 1 | H | Y | 1857 | NS3 | 355 |
| PA259249 | #1 | 5680 | C | T | 9.35 | Non-syn | 2 | S | F | 1858 | NS3 | 356 |
| PA259249 | a | 5680 | C | T | 1.55 | Non-syn | 2 | S | F | 1858 | NS3 | 356 |
| PA259249 | b | 5680 | C | T | 1.23 | Non-syn | 2 | S | F | 1858 | NS3 | 356 |
| MR766_∆E153-156_ | #4 | 7072 | G | A | 4.91 | Syn | 3 | A | A | 2322 | NS4B | 57 |
| MR766_∆E153-156_ | a | 7072 | G | A | 9.69 | Syn | 3 | A | A | 2322 | NS4B | 57 |
| MR766_∆E153-156_ | b | 7072 | G | A | 11.81 | Syn | 3 | A | A | 2322 | NS4B | 57 |
| MR766_∆E153-156_ | #4 | 7519 | G | A | 4.88 | Syn | 3 | L | L | 2471 | NS4B | 206 |
| MR766_∆E153-156_ | a | 7519 | G | A | 9.73 | Syn | 3 | L | L | 2471 | NS4B | 206 |
| MR766_∆E153-156_ | b | 7519 | G | A | 12.47 | Syn | 3 | L | L | 2471 | NS4B | 206 |
| PA259249 | #1 | 7644 | A | C | 1.30 | Non-syn | 1 | N | H | 2513 | NS4B | 244 |
| PA259249 | a | 7646 | C | T | 0.46 | Syn | 3 | N | N | 2513 | NS4B | 244 |
| PA259249 | b | 7646 | C | T | 0.44 | Syn | 3 | N | N | 2513 | NS4B | 244 |
| MR766_∆E153-156_ | #4 | 7729 | C | T | 4.06 | Syn | 3 | Y | Y | 2541 | NS5 | 25 |
| MR766_∆E153-156_ | b | 7729 | C | T | 1.66 | Syn | 3 | Y | Y | 2541 | NS5 | 25 |
| DakAr41524 | a | 8372 | A | G | 0.25 | Non-syn | 1 | T | A | 2756 | NS5 | 236 |
| DakAr41524 | b | 8372 | A | G | 0.19 | Non-syn | 1 | T | A | 2756 | NS5 | 236 |
| DakAr41524 | #2 | 8374 | A | G | 0.63 | Syn | 3 | T | T | 2756 | NS5 | 236 |

*The NGS data for replicates a and b of each strain were generated in the present study. All other sample NGS data were downloaded from the SRA database. #1: PA259249 SRR7879782; #2: DakAr41524 SRR7879856; #4 MR766_∆E153-156_ SRR7879861; #5: R103451 SRR7879832; #6 SRR7879847.

^Syn/Non-syn: synonymous vs non-synonymous substitution.
